# Serious Gaming During Multidisciplinary Rehabilitation for Patients With Chronic Pain or Fatigue Symptoms: Mixed Methods Design of a Realist Process Evaluation

**DOI:** 10.2196/14766

**Published:** 2020-03-09

**Authors:** Miel AP Vugts, Aglaia MEE Zedlitz, Margot CW Joosen, Hubertus JM Vrijhoef

**Affiliations:** 1 Tranzo Scientific Centre for Care and Wellbeing Tilburg School of Social and Behavioral Sciences Tilburg University Tilburg Netherlands; 2 Leiden Institute for Brain and Cognition Department of Health, Medical and Neuropsychology Leiden University Leiden Netherlands; 3 Department of Human Resource Studies Tilburg School of Social and Behavioral Sciences Tilburg University Tilburg Netherlands; 4 Department of Patient & Care Maastricht University Medical Center Maastricht Netherlands; 5 Panaxea Amsterdam Netherlands

**Keywords:** serious gaming, eHealth, chronic pain, medically unexplained symptoms, implementation, realist evaluation

## Abstract

**Background:**

Serious gaming could support patients in learning to cope with chronic pain or functional somatic syndromes and reduce symptom burdens.

**Objective:**

To realize this potential, insight is needed into how, why, for whom, and when it works in actual treatment circumstances.

**Methods:**

Following a realist approach, process evaluations were performed before, during, and after a two-armed, natural quasi-experiment (n=275). A group of patients with interfering chronic pain or fatigue symptoms received a short additional blended mindfulness-based serious gaming intervention during a multidisciplinary rehabilitation program. A control group only received the regular rehabilitation program. During two sessions before and one session after the experiment, expectations about serious gaming processes were discussed in focus groups with local care providers, implementers, and experts. Patients participated in a survey (n=114) and in semistructured interviews (n=10). The qualitative data were used to develop tentative expectations about aspects of serious gaming that, in certain patients and circumstances, trigger mechanisms of learning and health outcome change. Hypotheses about indicative quantitative data patterns for tentative expectations were formulated before inspecting, describing, and analyzing—with regression models—routinely collected clinical outcome data. An updated program theory was formulated after mixing the qualitative and quantitative results.

**Results:**

Qualitative data showed that a subset of patients perceived improvement of their self-awareness in moments of daily social interactions. These results were explained by patients, who played the serious game LAKA, as a “confrontation with yourself,” which reflected self-discrepancies. Important characteristics of serious gaming in the study’s context included innovation factors of relative advantage with experiential learning opportunity, compatibility with the treatment approach, and the limited flexibility in regard to patient preferences. Perceived patient factors included age and style of coping with stress or pain. Learning perceptions could also depend on care provider role-taking and the planning and facilitating (ie, local organization) of serious gaming introduction and feedback sessions in small groups of patients. Quantitative data showed very small average differences between the study groups in self-reported depression, pain, and fatigue changes (-.07<beta<-.17, all 95% CI upper bounds <0), which were mediated by small group differences in mindfulness (beta=.26, 95% CI .02-.51). Mindfulness changes were positively associated with patient involvement in serious gaming (n=114, beta=.36, *P*=.001). Acceptance of serious gaming was lower in older patients. Average health outcome changes went up to a medium size in patients that reported lower active coping with stress and lower pain coping before serious gaming. Mindfulness changes and gaming acceptance perceptions covaried with group structure and immediate feedback sessions after serious gaming.

**Conclusions:**

This study developed transferable insight into how and why serious gaming can facilitate additional learning about coping in order to reduce burdens of chronic pain or fatigue symptoms in certain patients and in actual treatment circumstances. Future studies are needed to continue the development of this fallible theory. Such research will further support decisions about using, designing, allocating, and tailoring serious gaming to optimize important patient health benefits.

**Trial Registration:**

Netherlands Trial Register NTR6020; https://www.trialregister.nl/trial/5754

## Introduction

### Background

Serious games could someday have substantial health impact by facilitating patients in learning to cope with chronic pain (CP) or functional somatic syndrome (FSS). A serious game is a kind of computer game that not only aims to provide *fun*, but also to inform, instruct, and modify [1]. In many patients with CP or FSS, functioning can improve with the moderation of psychosocial consequences or perpetuating factors of their symptoms [2,3]. Increasing evidence suggests that serious gaming can facilitate processes of change in health behaviors and their antecedents, as well as in mental health in patients of any age [1,4]. Randomized controlled trials with *stand-alone* serious gaming interventions have been reported [5,6]. The findings suggested clinically relevant mental health effects compared to waiting-list conditions, or an equivalent effect compared to *traditional* face-to-face delivery modes. However, complementary evaluation studies of strong internal and external validity were suggested [1,4]; they are needed to inform and legitimize the implementation of serious games on a large scale, which, assuming that development costs are high and variable costs low, is a plausible requirement to realize value for societies.

This study aims to develop theory with which future developers and implementers of serious games can improve their design and integration in regular health care settings. To achieve this, realist process evaluations were embedded into a quasi-experiment with the serious game LAKA.

### Burdens of Chronic Pain and Functional Somatic Syndromes

CP is pain, with or without a specific organic cause, that persists longer than a usual 3- to 6-months of organic recovery [3]. An FSS is characterized by a persistent pattern of bodily symptoms that cannot be sufficiently pathologically explained after adequate physical examination [2]. Close to 1 in 5 adults has intense pain (ie, rating 5/10 or more, for at least 6 months) [7-9]. FSSs characterized by irritable bowels (11%), chronic fatigue (1%), tension headache (2%), or tinnitus (10%-15%) are prevalent as well [10-12]. The severity and chronicity of lower back pain, which is the most common symptom of both CP and FSS, may be associated with more functional disturbance during people’s lifetime than any other disease [13].

CP and FSS often come with psychological or social burdens. Comorbid mental disorders were found in 35% of patients with CP [14]. Similarly, substantial minorities of patients with FSS also have another FSS or a psychological disorder [15,16]. Compared with individuals without pain, those who experience more than 3 months of disabling pain (7.4%) are about six times more likely to be absent from work and two times more likely to visit a doctor [17]. In addition, economic costs of sick leave and early retirement exceed the total amount of medical expenditures associated with both CP and FSS [18-20].

### Biopsychosocial Management

For managing burdens of CP and FSS, biopsychosocial approaches are considered effective [2,3]. Meta-analyses show positive effects for physical and emotional functioning of various treatment options (ie, medication, complementary medicine, psychological therapy, or multidisciplinary rehabilitation) that are small or medium sized, at most [21-28]. Recommended treatment includes steps of conservative medication, psychotherapy, and physiotherapy, and a multi- or interdisciplinary rehabilitation program when previous treatments do not suffice [2]. Multidisciplinary rehabilitation programs vary in content but commonly include medical, psychological, physical, and occupational interventions [21,26]. New knowledge, with which treatment effectiveness for different CP and FSS patients may be improved, comes from research into the following: (1) the biological mechanisms of specific symptom patterns [29] and (2) how *adequately* (eg, feasibly or cost-effectively) already understood biopsychosocial mechanisms are targeted by certain treatment approaches, strategies, and modes of delivery [30,31]. Regarding the latter, current interest goes to the potential of facilitating intervention with computer technology [32,33].

### How and Why Biopsychosocial Approaches Work, for Whom, and When

CP and FSS conditions are diverse, but a common, partial, explanation is given by the sensitization of the central nervous system [34]. Biopsychosocial approaches target the following: (1) peripheral systems with pharmacotherapy (ie, *bottom-up* approaches) as well as (2) cortical brain systems with neuroscience education, cognitive behavioral therapy, and exercise (ie, *top-down* approaches). Changes in coping with stress and pain, avoidance beliefs, rumination, acceptance, or catastrophizing mediate intervention effects on experienced physical and mental functioning [35,36]. An approach for changing coping responses is to promote individual mindfulness (ie, self-awareness, self-regulation, and self-transcendence) in response to adverse experiences (eg, negative emotions or physical sensations) [37]. Another approach is to restructure particular cognitive antecedents of pain-related experiences and behaviors [38-41]. Whether and how these approaches differ with respect to underlying change mechanisms and outcomes has been debated [38,39].

Literature about patient factors for effectiveness provide limited evidence to inform practice. Some studies claimed that neither demographic nor psychological differences between patients predict clinically important variation in treatment effects [42,43]. Other studies, however, stressed that fear-avoidance beliefs, pain acceptance, or depressive symptoms can predict treatment gains to some extent and should therefore be targeted early or additionally in treatment [44-47].

More insight is also needed about the characteristics of interventions and treatment contexts that are responsible for varying intervention effects. Meta-analytic results showed that computer-based intervention (eg, delivery over the Internet) can provide modest chances for symptom reduction, similarly to face-to-face group therapy of similar content [30]. It also showed uncertainty about the transferability from results in self-selecting participants to wider populations and settings. Researchers have been repeatedly suggesting that better explanations of varying effectiveness levels between studies requires *contextualization*; for instance, consideration of (1) factors such as fidelity of implementation, program compositions, and comparisons and (2) interdependencies and interactions between such factors [31,44,47-49].

### How and Why Serious Gaming May Work, for Whom, and When

For serious games, there is a similar need for theories of what works how, for whom, and when [50,51]. Games or virtual reality have motivational qualities that may support the extinction of phobias, distraction from pain, repetitive physical or cognitive training, or learning about cognitive antecedents (eg, self-efficacy) of health behavior [52-54]. Gaming features, including stories and interactivity, may strengthen behavioral change processes with engagement, intrinsic motivation, positive affect, and sense of presence when processing educational content [52,55]. Various studies showed that effects of serious games can vary with intervention factors (eg, participatory design and duration) and users (eg, gender, age, intelligence, and gaming experience) [1,51,56]. Individual experiences of serious gaming may be difficult to predict and may be context dependent [57,58]. Debriefing is a way of dealing with such variation by leveraging recipient experiences after serious gaming for transferrable individual learning results as intended [59].

### Effectiveness of Serious Gaming During Treatment for Adults With Chronic Pain or Functional Somatic Syndrome

To the best of the authors' knowledge, the first outcome evaluation of serious gaming for reducing disease burden in patients with CP or FSS was reported [30,60]. The effect of serious gaming was studied in a quasi-experiment with patients with mostly chronic musculoskeletal pain and psychosocial problems. A comparison was made of symptom changes during a regular multidisciplinary rehabilitation program (100 hours) between (1) an intervention group that received an additional *blended* intervention with the game LAKA (4 extra hours) and (2) a control group that received no serious gaming. A very small acceleration of physical and emotional symptom reduction was attributed to serious gaming: a standardized regression coefficient for the group × time effect of 0.12 [60]. This effect size estimate corresponds with a comparable estimate from an earlier meta-analytic subgroup analysis (ie, a 0.13 standardized average difference in short-term behavioral or mental health outcomes between groups following multi-component interventions *with* and *without* serious gaming) [1]. This suggests that a couple of hours of serious gaming as part of treatment does not provide a general clinically relevant benefit, per se, but can add to the effectiveness of the treatment as a whole. Plausibly, serious games and other computer applications for CP or FSS patients reach stronger, average effects when delivered as stand-alone interventions in other circumstances (eg, when compared with no active treatment) [1,30].

### Existing Theories to Inform Intervention Theory

Existing theories on related topics provide a starting point for building transferrable insights into how and why features of a serious gaming could work in certain contexts for patients with CP or FSS (see Textbox 1). Changes of rehabilitation outcomes due to serious gaming are potentially explained with theories about health behavior, including mindfulness [37], relational framing [61], psychological well-being [62], or self-discrepancy theory (SDT) [63]. Why serious gaming features could be a distinctive trigger for learning has been explained with theories about motivational and affective responses to virtual or computer gaming environments [52,64]. Other potentially applicable theories described a comprehensive range of potential context factors for implementing health care innovations [65,66]. More research upon which theory building rested is summarized in Textbox 1 [67-71].

**Candidate theories for explaining effects of serious gaming: potentially applicable, formal explanatory frameworks.**

Rehabilitation mechanisms:

- One theoretical model aligned particularly well with initial developer ideas about the intervention mechanisms [37]. Mindfulness covers several mental training practices and their consequences for cognitive functioning. These consequences are described by self-awareness, self-regulation (ie, the ability to change one’s own behavior), and self-transcendence (S-ART) (ie, prosocial characteristics). Three subtypes of practices are distinguished. Two of these are explicitly instructed and encouraged in LAKA (ie, focused attention and open monitoring), and one (ie, ethical enhancement) was integrated into immersive simulation tasks (ie, virtual social interactions or encounters). The S-ART model describes how these three types of practices influence the brain networks that support intention and motivation, attention regulation, emotion regulation, extinction and reconsolidation, prosociality, nonattachment, and decentering. From this theory, it is plausible to suggest that the features of a game such as LAKA can support an introduction to mindfulness practice for novices, rather than a tool for long-term exercise.
- Relational frame theory [61] underlies third-wave cognitive behavioral change approaches, such as acceptance and commitment therapy. It explains the harmful outcomes of cognitive fusion (ie, fusing thoughts with reality) and experiential avoidance (ie, attempts to avoid thoughts, feelings, memories, physical sensations, and other internal experiences) in functional contexts.
- Eudaemonist approaches to psychological well-being [62]. The rehabilitation approach, and the intended role of serious gaming in it, is to direct attention toward participation in social roles and psychological well-being rather than to necessarily change particular symptom-related cognitions or behaviors. With tasks in LAKA, players are challenged—or enabled to experiment with—vicarious prosocial behavior. Intrinsically or autonomously motivated prosocial behavior is known to promote psychological well-being [67].
- Self-discrepancy theory (SDT) can also be a useful explanatory framework for serious gaming effects. This was also hinted at in previous pilot results [68]. SDT predicts particular affective states (eg, dissatisfaction, disappointment, and agitation) from the degree and nature of discrepancy between a person’s actual self-state and a self-script (ie, ideal or ought to states from own or others' perspective) [63]. Self-discrepancies also partially explain comorbidity of depression and chronic pain (CP) [69,70]: when pain or fatigue symptoms become chronic, self-scripts conditional on the absence of those symptoms may be a source of emotional disturbance and maladaptive activity patterns. SDT was used to predict functional improvement in CP patients by motivating pursuit of possible selves or perceptions, such as interest, approval, or acceptance, that are not actually conditional to physical symptoms [71].

Mechanisms of computer gaming:

- SDT could also play a role in understanding responses to computer gaming. Previous research showed that perceived opportunity for realizing ideal selves drives engagement in computer games, and especially in individuals with larger actual-ideal self-discrepancies [64].
- In addition, various processes of motivation, affect, and immersion during serious gaming were considered to potentially strengthen learning or behavioral change effects [52].

### Study Objectives

This study primarily aims to provide in-depth information to recipients, developers, evaluators, and implementers on contexts in which certain patients with CP or FSS can use features of serious gaming in ways that result in clinically important health benefits. The overall research question is as follows: (1) *How*, (2) *why*, (3) *for whom*, and (4) *when* does serious gaming lead to learning and health outcome change? Accordingly, the objectives are enhanced propositions about the following:

1. How do blended and mindfulness-based serious gaming interventions lead to additional learning and health outcome improvement?
2. Why do serious gaming interventions lead to additional learning and health outcome improvement? What generic, formal theory supports the explanation?
3. For which patients with CP or FSS are serious gaming interventions feasible and effective with respect to additional learning and health outcome improvement?
4. Under what circumstances of implementation in a regular multidisciplinary rehabilitation treatment are serious gaming interventions feasible and effective with respect to additional learning and health outcome improvement?

## Methods

### Process Evaluation Approach

The process evaluation uses a realist evaluation approach [72]. Empirical process evaluations are the preferred method to gain a thorough understanding of which intervention features contribute to clinically relevant benefit, how, why, for whom, and under which *complex* circumstances in real health care contexts [73,74]. Such insights can benefit future choices of development, evaluation, and implementation (eg, selective allocation, tailored design, and rollout in other settings). Realist principles can guide theory-based and contextual, sensitive, process evaluations of complex *programs* or *interventions*—terms are used interchangeably [73,75].

A *program theory* is developed on the basis of empirical evidence that indicates whether initial ideas are supported or must be refuted, extended, or refined. Program theories are built up from configurations (C) of intervention characteristics (I) in context (C) triggering a mechanism (M) that leads to certain outcomes (O) (ICMO configurations or ICMO-Cs). An intervention is something new to, or extracted from, a pre-existing situation, certain aspects of which interact with the intervention to elicit causal effects (ie, context). A *mechanism* is an underlying (ie, invisible) causal force of empirical events, that is, not a description of successive events under counterfactual situations [76]. Mechanisms have often been framed as the reasoning with which recipients respond to the resources of interventions [77], but ideas about what they are can vary with the topic of interest. The ICMO-C is also applied to conceptualize on a *middle range* of abstraction for “dealing with different spheres of behaviors and structures to transcend sheer description” [76]. Realist approaches use induction, to discover regularities in empirical phenomena, in alternation with deduction, to formulate expected observable consequences of abstract formal theories, for inferring the *best* explanation of observable outcome patterns (ie, *abduction*) [78].

### Initial Program Theory

An initial ICMO-C was formulated by taking assumptions from several of the introduced theories on related topics (see Textbox 2 as well as Multimedia Appendix 1 for a comprehensive overview). In addition, it was expected that serious gaming would be more generally accepted and better adhered to after improving the delivery modes of serious gaming in the treatment context, as suggested by previous pilot study results [67].

**Initial intervention-context-mechanism-outcome (ICMO) configuration.**

Intervention in context:

Mindfulness-based serious gaming delivered in blended form (ie, a combination of computer gaming and face-to-face guidance) as a standard component during multidisciplinary rehabilitation (ie, intervention in context) is accepted and adhered to by patients (ie, feasibility mechanisms) with a complex chronic pain or functional somatic syndrome condition (ie, patient in context).

Mechanism:

Serious gaming, as such, can provide complementary features, such as sounds, visuals, stories, or covert learning strategies, that trigger distinctive experiential, affective, or motivational qualities. This can include the degree or valence of affect or sense of presence (ie, gaming mechanism). These experiences strengthen learning results with respect to *mindfulness*, coping flexibility, or psychological well-being (ie, rehabilitation mechanism or intermediate outcome).

Outcome:

The learning results subsequently contribute to reductions in physical and emotional symptoms of pain intensity, fatigue, and depression (ie, rehabilitation outcome).

### Mixed Methods Design

The process evaluation is designed as an embedded, two-armed, natural, quasi-experimental, mixed methods study [66]. It was carried out by an integrated team of researchers, who are trained in various quantitative and qualitative methods. Priority was given to quantitative methods for investigating patterns in routine clinical patient outcome assessments. Patients were recruited who were following a standardized 16-week multidisciplinary rehabilitation program, of 100 hours on average, in one of four treatment sites of a single Dutch rehabilitation center. At two sites, an additional 4-hour *blended* intervention with the mindfulness game LAKA was provided during the second half (weeks 9-12) of the rehabilitation program (ie, intervention condition). In the other two sites, gaming was not offered (ie, control condition). Measurements were routinely taken at baseline (t0), at an intermediate time point after 8 weeks of treatment (t1), and posttreatment after 16 weeks (t2). Nonintrusive qualitative data collection took place before, during, and after the experiment. Analyses of qualitative data for formulating hypotheses preceded the inspection and analyses of the quantitative outcome data (see addendum to the registration in the Netherlands Trial Register, NTR6020). All steps from the qualitative and quantitative research are presented in Table 1.

The alternative hypotheses formulated in step 2 specify certain patterns of linear relationships between variables that operationalize outcomes and characteristics of the intervention in context. Such statements were listed, a priori, to strengthen support, or refutations, of tentative ICMO elements. An ICMO-C gains support, or refutation, with quantitative results that should or should not be considered very unlikely under the assumption that the ICMO element is superfluous, as follows:

1. The effect of serious gaming on patient outcomes (ie, depressive mood is mediated by change in learning results, such as mindfulness).
2. Sense of presence and positive affect during serious gaming are positively related with changes in learning results and health outcomes.
3. Learning results, subsequent health outcome change, or game acceptance perceptions vary with patient-level factors of age, coping (ie, an active style and existing use of various alternate ways of pain coping), and room for health improvement (ie, psychological symptoms).
4. Learning results, subsequent health outcome change, or game acceptance perceptions vary with differences in the organization of sessions (ie, timing, presence of other patients, care providers, and changes of intervention in context over time).

The Adjudicating Formal Theory and Formulating Hypotheses section explains the ICMO elements to which the hypotheses relate.

**Table 1:** Steps of recruitment, data collection (steps A-G), and analysis (steps 1-5).

Protocol steps	Research activity
A (data collection)	Recruit stakeholders and perform focus group interviews (two sessions)
B (data collection)	Recruit patients
C (data collection)	Collect post-serious gaming feedback from professional and patient users
D (data collection)	Purposively select patients for semistructured interviews
E (data collection)	Perform semistructured interviews with patients
F (data collection)	Perform stakeholder focus group interview (third session)
1 (analysis)	In iteration with steps E and F: Code intervention (I), context (C), mechanism (M), and outcome (O) elements in all the qualitative information Describe ICMO relationships per individual patient interview Compare patient-level findings with focus group data collected from other stakeholders
2 (analysis)	Interpret mechanisms on the basis of formal theory (ie, adjudication) Formulate quantitatively testable hypotheses before outcome inspection
G (data collection)	Retrieve quantitative data from patient records
3 (analysis)	Describe quantitative data as a means to triangulate the qualitative results
4 (analysis)	Test hypotheses with statistical models
5 (analysis)	Mix the results of different methods: summarize how they are interpreted to support, refute, refine, or extend initial expectations Construct summary ICMO configurations (*middle-range* theory) based on findings of this study Propose a transferable program theory after comparison of findings from this study with those of previous studies

### Setting and Participants

The setting and participants were fully described in the outcome evaluation report [59]. Treatment and control sites had similar protocols, sizes, histories, and absence of disruptive activities during the study period. Patients with a physicians' indication of eligibility for multidisciplinary rehabilitation received informed consent from a familiar care provider soon after the second part of rehabilitation treatment started. Participation included additional data collection for the study and permission to process codified, routinely collected, clinical patient data. A total of 275—156 intervention group and 119 control group participants—out of the 329 eligible patients participated (83.6%). Patients in the sample were, on average, 44 years of age (SD 11.3, range 18-67). The proportion of females was 69.8% (192/275). Almost half of the patients (134/275, 48.9%) reported a symptom duration of over 2 years. Patients (N=275) mostly had musculoskeletal pain and concomitant psychosocial problems. The findings of the outcome evaluation suggested no confounding influences by treatment site or patient baseline variables that differed, statistically significantly, in mean levels between the study groups.

Other participants included care providers (ie, three psychologists and one physiotherapist), local managers who were responsible for providing the serious gaming intervention on site, and stakeholders with relevant expertise in information and communication technology (ICT), serious gaming, rehabilitation medicine, health psychology, and spiritual counseling. All were familiar with the setting and provided informed consent on the participation in focus group sessions.

### Interventions

Features of the multidisciplinary treatment and additional serious gaming intervention offered to the intervention group were previously reported in detail [66]. A short description of LAKA is given in Textbox 3. The clinic facilitated tablet computers, suitable rooms with Wi-Fi connections, and the automated planning of four 1-hour sessions, for 1-6 patients simultaneously, in connection to regular therapy hours. Three psychologists and a physiotherapist (two per site) provided support during the first (ie, introduction) and fourth (ie, debriefing) sessions. Topics of debriefing were technology acceptance, play experiences, and *learning transfer* to daily life. Access to LAKA during the second and third sessions was provided on site by local staff members. In addition, patients could play LAKA at home on a tablet computer with an Internet connection. This connection made it possible to download LAKA via an app store and to safely exchange data with the rehabilitation center for access to the app, storage of progress, and feedback on performance. All patients were expected to attend a debriefing session after completing the game at least once: this took 2.5 hours on average. This intended *dose* was based on what had been a natural amount to patients during a feasibility study [68].

**Short description of the serious game LAKA.**

LAKA challenges patients to take the role of a virtual character (ie, avatar). The gameplay includes prompts for monitoring and evaluating *satisfaction* about selected *responses* in virtual social encounters. These optional responses are descriptions of implementation intentions for acts, phrases, and postures in social interaction scenarios that players can select for their avatar. This avatar represents themselves on a virtual trip around the world. Optional responses—five at each moment—vary in the degree of correspondence with the principles of *generosity*, *moral discipline*, *patience or forbearance*, and *enthusiastic perseverance*. Each selected response has salient (eg, emotional expressions) and realistic (ie, not predictable or moralistic) consequences for the avatar. These consequences are intended to evoke reflection. Neutral to positive indirect performance feedback reinforces the monitoring task. Moreover, scenarios prompt instructions for 3-minute focused attention or open monitoring (ie, meditation) exercises. Encouragement stimulates users to repeat the exercises at any convenient moments in daily life.

### Qualitative Data

Interviews and feedback surveys contained open-ended questions, which were intentionally free from theoretical preconceptions. Such open-ended questions were posed first to give space for explanations of initial reactions, including unexpected ones. Topics and additional follow-up questions for interviews were based on the existing theoretical frameworks on related topics (see Introduction section). The prioritization of topics varied with the expected knowledge areas of the participants [79]. Participating patients were considered to be most aware of outcomes and characteristics of the intervention in context, while care providers knew most about rehabilitation mechanisms.

Focus groups were first held with stakeholders who represented various roles and areas of expertise. Participants prepared for the focus groups by playing LAKA and reading pilot study results and adapted intervention specifications. At the beginning of the session, the research questions were presented for collecting local experts' initial expectations. In *part two*, care providers and implementers built upon shared expectations for specifying local implementation procedures in further detail. The second and third focus group interviews were held with two care providers who worked at the same intervention site. At the beginning of the second focus group, initial expectations about intervention effects on rehabilitation mechanisms were explained. During the natural experiment, care providers also shared feedback informally with MAPV. The third focus group with care providers first generally addressed their postexperimental experiences. These were about how recipients responded to the intervention in the context, including how those responses were shaped by care providers' own reactions. The discussion was enriched by sharing tentative qualitative results (ie, parts of tentative program theory).

The first source of qualitative patient information consisted of the given responses on two open-text requests after finishing playing LAKA: “Please describe in your own words what you experienced when you played LAKA” and “Please explain how the LAKA sessions, according to you, will contribute to your daily life.” Furthermore, semistructured interviews were held with patients. These interviews were held with (1) intervention group patients who were purposively selected by differences in outcome expectations and (2) control group participants with similar baseline characteristics as interviewed intervention group patients. Interviews took place when patients had completed their rehabilitation program. Main topics of each interview were *what worked and how*, in order to change courses of symptoms and learning in the specific situations of the patient throughout rehabilitation (eg, “Could you explain how your health status developed throughout the rehabilitation program? What aspects of the rehabilitation program really made a difference for you? What was the role of serious gaming, if any, assuming differences occurred? What aspect of the game contributed like that?“). A data saturation point was reached when no new information emerged by asking patients about three *good* and three *bad* things about serious gaming. The more initial responses from recipients were already collected and analyzed, the more often MAPV shared an interpretation of a fragment in order to question its accuracy. If potentially relevant topics for the development of program theory were not addressed at the initiative of patients, they were sometimes introduced with questions that referred more directly to specific theory-based expectations (eg, ”Were you concentrating while playing LAKA?“).

### Quantitative Data

The initial program theory informed the collection of quantitative data sources. Table 2 lists the times of assessment and the internal consistency information of all quantitative measurements that were processed for hypotheses testing. Health outcomes were measured with validated questionnaires on depressive mood, pain intensity, and fatigue as part of the routine clinical data collection [68,80-82]. *Learning result* was measured with a mindfulness questionnaire, of which unpublished psychometric results gave support for being a valid measure (see Multimedia Appendix 2, which contains information in support of a good internal consistency, low social desirability, and patterns of association with other constructs that are similar to those of other validated mindfulness questionnaires) [83,84].

For additional indications about potential mechanisms, a short *postgaming* feedback questionnaire with complementary scales was added to the routine clinical measurement system. This postgaming feedback questionnaire included Likert-scale items on sense of presence (ie, involvement and realism subscales) [85], positive and negative affect [86], and patient acceptance perceptions with regard to the serious gaming intervention [87,88].

Indicators of patient factors included clinical data about demographic, health status, and coping [89,90] variables as measured at the latest available time point before serious gaming. Classification norms from manuals of the questionnaires were used to divide patients over subgroups. Log data about the circumstances of the intervention in context were disaggregated to the patient level (see Table 2). This includes dummy variables for indicating each intervention site, the care provider who provided debriefing sessions, and the social *structure* of debriefing sessions: a care provider with a patient alone, a dyad, or a group. Moreover, differences between dates indicated coincidental variation by central planning and general changes of serious gaming sessions in time.

**Table 2:** Overview of quantitative data.

Classification as intervention, context, mechanism, or outcome, and theoretical construct		Variables (operationalization)	Time of measure (Cronbach alpha)
**Rehabilitation outcome (health outcomes)^a^**			
	Depressive mood	Symptom Checklist-90 (SCL-90) depression subscale [80]	t0^b^, t1^c^, t2^d^ (.91, .91, .91)
	Pain intensity	Numerical rating scale (current) 0-100 [81]	t0^b^, t1^c^, t2^d^ (N/A^e^)
	Fatigue	Checklist of individual strength [82]	t0^b^, t1^c^, t2^d^ (.86, .95, .96)
**Rehabilitation mechanism, serious gaming outcome, learning result**			
	Mindfulness	Sums of scores of three subscales for *mental stability*, *forbearance*, and *enthusiastic perseverance* behavior (eg, “Also in a turbulent environment, I can concentrate well” and “I remain patient until I see the solution”)	t0, t1, t2 (.94, .95, .95)
	Gaming performance	Standardized values for responses (ordinal scales 1-5)^f^	Log data (N/A)
**Serious gaming feasibility outcome, implementation fidelity**			
	Adherence	Progress: number of encounters completed	Log data (N/A)
		Attendance of serious gaming sessions	Log data (N/A)
**Serious gaming mechanisms**			
	Involvement	Igroup Sense of Presence Questionnaire [85]	Postgaming (.76)
	Realism	Igroup Sense of Presence Questionnaire [85]	Postgaming (.69^g^)
**Experiential qualities**			
	Positive affect	Positive affect scale: Positive and Negative Affect Schedule (PANAS)-short form [86] (1-5)	Postgaming (.86)
	Negative affect	Negative affect scale: Positive and Negative Affect Schedule (PANAS)-short form [86] (1-5)	Postgaming (.80)
**Serious gaming feasibility mechanism, intervention**			
	Game acceptance perceptions	The average score was taken from the following Likert-scale items^h^: From the UTAUT2^i^ questionnaire [87]: Perceived usefulness (“By following the LAKA module, I could achieve my health goals more quickly”); Perceived ease of use (“Learning how to use LAKA was easy for me”); Perceived trust (“The LAKA module offers services in my best interest”); and Perceived enjoyment (“Following the LAKA module was enjoyable”). From the EgameFlow questionnaire [88]: Clear goals (“Overall goals of LAKA were presented clearly”); Challenge (“LAKA provides different levels of challenges that tailor to different players”); and Perceived learning (“The LAKA module increased my knowledge”).	Postgaming (.83)
	Outcome expectations	“Use the following slider (0-10) to indicate to what extent you expect that the LAKA sessions contribute to your daily life”	Postgaming (N/A)
**Patient factors**			
	Demographics^j^	Age in years (>45=high)	t0 (N/A)
		Female or male	t0 (N/A)
		Socioeconomic status by neighborhood	t0 (N/A)
	Coping with stress and pain	Utrecht Coping List [89]. Active coping scale (>20=high)	t1 (.81)
		Pain Coping and Cognitions List [90]. Pain coping scale (low=a standardized residual score <-1 after regression on relevant factors: gender with male as reference [beta=.14, *P*=.03] and socioeconomic status [beta=.16, *P*=.02])	t1 (.85)
	Psychological distress	SCL-90. Total score (>132 is high or very high)	t1 (.97)
**Intervention in context**			
	Setting (treatment site)	Four dummy variables: Site 1; Site 2; Site 3; and Site 4.	t0 (N/A)
	Intervention	Identifiers of intervention versus control group (ie, dummy); intervention (site 1-2) versus control (site 3-4)	t0 (N/A)
	Inner setting (provider)	Five dummy variables for the care provider from whom debriefing was received: Care provider 1 vs other; Care provider 2 vs other; Care provider 3 vs other; Care provider 4 vs other; and Stand-in care provider.	Log data (N/A)
	Implementation (development of quality over time)	Introduction session dates	Log data (N/A)
	Planning	Differences in days between the following: Intermediate outcome assessment and introduction session; Introduction and debriefing sessions; Debriefing and posttreatment assessment; and Last time playing LAKA and debriefing (short <3 days).	Log data (N/A)
	Social *structure* (of serious gaming sessions)	Three dummy variables on the numbers of patients who were simultaneously attending debriefing sessions (as determined by planning): The patient was in a group (2 or more other patients were present); The patient was alone (ie, one-on-one debriefing by a care provider); and There was 1 other patient (dyad).	Log data (N/A)

^a^This report omits a primary outcome measure for pain catastrophizing, as previous outcome evaluations found no indication that variance in this outcome was attributable to serious gaming.

^b^t0: at baseline.

^c^t1: at intermediate time point after 8 weeks of treatment.

^d^t2: after 16 weeks of treatment.

^e^N/A: not applicable.

^f^Log data included log ins, selections made by patients for their avatar (ie, gaming performance), and last completed encounter. These were automatically collected through a secure Internet connection for saving progress and providing performance feedback. Elucidating whether game scores are valid indicators of learning is a technical challenge warranting focused research attention.

^g^Internal consistency of this scale was considered insufficient; therefore, sensitivity analyses were performed in which scores were replaced by the average of three scores, excluding one item, for which Cronbach alpha=.73. There were no relevant changes of the results.

^h^These items were selected because they had the strongest factor loadings within the scales to which they belong as established in a pilot study [68]. Together, the items formed an internally consistent scale.

^i^UTAUT2: second generation of the Unified Theory of Acceptance and Use of Technology.

^j^More information was retrieved for the evaluation project. Information on demographics that are only processed for the outcome evaluation and not for the present process evaluation, specifically, were excluded.

### Qualitative Analyses (Steps 1 and 2)

All interview texts (ie, verbatim transcripts) were coded by MAPV in ATLAS.ti, version 7.5.16 (Scientific Software Development GmbH), using sensitizing concepts for intervention, context, mechanism, and outcome unless new codes were needed to cover *unexpected* statements. The first four patient interviews were also independently coded by MCWJ (two interviews) and AMEEZ (two interviews). Unresolved differences were discussed with HJMV. It was counted how often codes for open-feedback text fragments recurred to get a sense of the relative importance of various themes and coverage of those by the interview data.

In step 1, ICMO relationships were determined per patient interview when explicit statements were found about relationships between positive outcome expectations, mechanisms, and the intervention in context (see Multimedia Appendix 3 for illustrations). In addition, intervention or context factors were coded as *barriers* when they were explicitly related to suboptimal, weak, or absent activation mechanisms or outcomes. Tentative ICMOs, documented down in theoretical notes, summarized regularities that were found when comparing individual patient results with each other and with the qualitative data that were obtained from other stakeholders. Theoretical notes formed the basis for regular bilateral or team discussions that continued during the next step.

In step 2, decisions were made about which formal theoretical perspectives were most suitable for explaining the qualitative data about intervention mechanisms. This was a starting point for adding, actually resuming, a deductive process to specify indicative patterns of quantitative data (ie, observable effects) of tentative ICMOs. Hypotheses were formulated that were testable with the available (ie, uninspected) quantitative data. Formulations of hypotheses had to take into account the limitations of the sample size, avoiding too many or too complicated hypotheses, and a lack of possibilities to add new quantitative measurement instruments at that time. The research protocol was ethically assessed before data collection began.

### Quantitative Analyses (Steps 3 and 4)

The ggplot2 package from R, version 1.1.463 (The R Foundation), was used to visualize levels of change in health outcomes, change of mindfulness, and game acceptance levels between subgroups of patients in context. Provisional classifications of *favorable* or *not favorable* were made with combinations of variables about the following: (1) whether a patient received the serious game intervention and how the implementation went, individually, and (2) his or her pre-existing characteristics.

All prediction models were executed in SPSS, version 24.0 (IBM Corp), using applications of the ordinary-least-squares estimation algorithm. All models were based on complete cases. All variables, except for dummy variables and dates, were centered or standardized before inclusion in statistical models. Outcome change indicators are standardized residual scores after regression of postintervention (t2) scores on preintervention (t1) scores. Decisions on the null hypotheses are informed by two-sided *P* values and 95% CIs. Various sensitivity analyses were performed to indicate the robustness of the methodological choices, including missing data handling. These analyses are discussed among the strengths and limitations.

**Figure 1:**
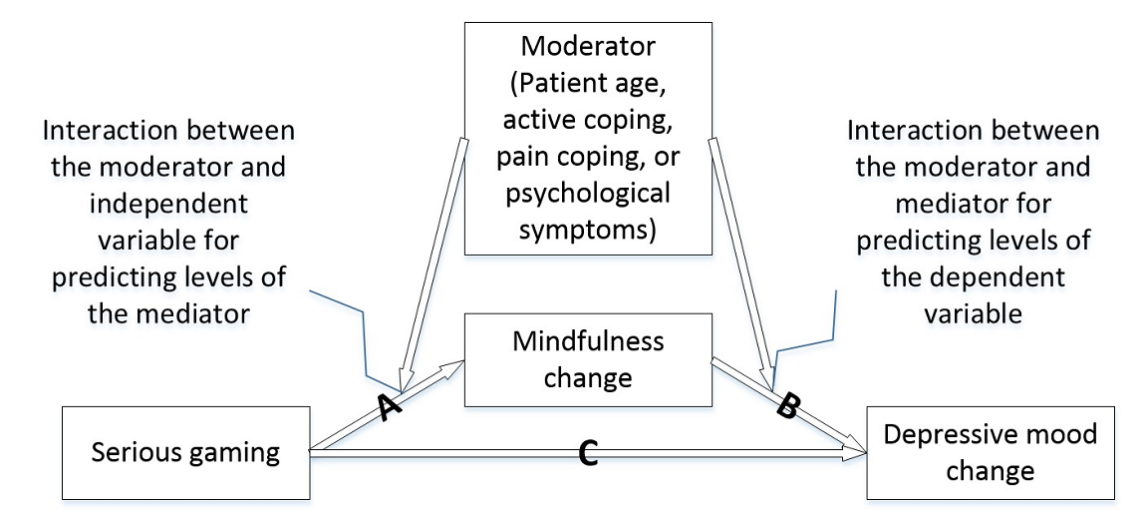
Illustration of the moderated mediation models.

The first type of procedure applied Hayes’ PROCESS macro, version 2.16, for SPSS to investigate hypotheses 1 and 3 [91]. These procedures calculate CIs using a nonparametric bootstrap approach that, by default, generates 5000 samples. Hypothesis 1 was investigated by fitting models of *type 4*. These models specified estimations for effects of study group on changes of the three health outcomes as mediated by mindfulness changes. Mediation models were extended to moderated mediation models of *type 58* for testing hypothesis 3 (see Figure 1). These models calculated the following: (1) parameters for the interaction between the moderating patient factors and study group for predicting levels of the mediator (ie, mindfulness change), (2) parameters for the interaction between the moderating patient factors and the mediator for predicting levels of the health outcomes, and (3) an index of moderated mediation assessing differences in the total indirect effects between two patient subgroups.

Secondly, multivariable linear regression models were used. A model with mindfulness as the dependent variable, and the subscales of sense of presence and affect as the independent variables, provided relevant test statistics to decide on hypothesis 2. A similar regression model, with game acceptance perceptions as the dependent variable, and patient factors (ie, continuous) as the independent variables, informed, in part, hypothesis 3. Three sets of regression models were used to test whether the variables for *intervention* or *context* factors, as the independent variables, affected the variables of (1) game acceptance, (2) mindfulness change, or (3) depression change. To prevent too many factors from being added to a single model, *intervention* *in* *context* variables were added and removed one by one after controlling for patient age and active coping style. Only the planning variables (ie, date differences) were added and removed simultaneously to isolate unique influences, taking interdependency into account.

### Mixing the Results of Different Methods (Step 5)

The final middle-range ICMO-Cs were constructed by assuming elements of the initial ICMO-Cs, refinements, and extensions based on the results from steps 1 and 2 that were not refuted based on the quantitative results from steps 3 and 4. The final conclusions are based on comparisons of the results, with respect to the elements of the ICMO-C, between this study and previous studies.

## Results

### Participants

Postgaming feedback data were collected from 114 out of 156 (73.1%) patients of the intervention group. Nonresponders (mean 40.6 years old, SD 11.7, *P*=.02) were relatively younger than responders (mean 45.5 years old, SD 11.2) and completed, on average, 1.5 more encounters (15.1 versus 13.6) in the game LAKA (*P*=.02). No other statistically significant differences were observed between these groups. Two care providers participated in the first focus group, together with a rehabilitation physician and serious gaming expert, a spiritual counselor, an executive and ICT expert, and two managers. The four care providers—three psychologists and one physiotherapist—who regularly facilitated introduction and debriefing sessions participated in the second and third focus groups. The 8 interviewed patients from the intervention group reported differences in the conditions in which they received serious gaming, their demographics, intervention experiences, and outcome levels (see Table 3). All patients reported having at least completed a secondary education. Recruitment of control patients was stopped after two, short, 10-15-minute interviews, because the interviews did not provide useful data.

**Table 3:** Characteristics collected from interviews with patient participants from the intervention group.

Characteristic	Patient number							
	1	2	3	4	5	6	7	8
Age in years	51	34	46	45	55	47	56	55
Gender (female=1; male=0)	1	0	0	1	0	1	0	1
Highest education level^a^	MV^b^	2	3	3	2	3	3	MV
Encounters completed (range 0-28)	14	15	24	28	12	16	16	1
Site ID	1	2	2	2	1	1	2	2
Symptom Checklist-90 (SCL-90) depression score (range 16-80): t1 to t2^c^ decrease	2	4	17	7	17	32	12	1
Mindfulness (range 49-245): t1 to t2 increase	6	10	24	11	27	35	28	43
Perceived outcome (range 0-10)	9	8	7	6	5	5	1	0
Negative affect (range 0-20)	0	3	2	5	10	1	2	20
Positive affect (range 0-20)	17	18	11	19	6	12	3	0
Involvement (range 0-6)	6	4.5	4.5	4	3	4.75	2.25	3
Realism (range 0-6)	6	4	2.25	3.25	3.5	2.75	3	0
Group size debriefing (range 1-6)	1	5	2	1	6	5	2	3

^a^Highest education level: secondary (ie, high school level) as highest=2; tertiary (ie, college or university level) as highest=3.

^b^MV: missing value.

^c^t1: intermediate time point after 8 weeks of treatment (ie, before serious gaming); t2: after 16 weeks of treatment.

### Results of Coding Qualitative Materials (Step 1)

#### Learning Results

In all interviews except for one, patients mentioned barriers—intervention or context—for serious gaming outcomes. ICMO-Cs emerged from the six out of eight interviews with patients that had moderate-to-high outcome expectations: the scores were 5-9 out of 10. Among all 114 survey respondents, 61 (53.5%) had at least moderate outcome expectations. Statements about outcomes were coded using sensitizing concepts about mindfulness (ie, a heightened self-awareness about certain dysfunctional mental states when reacting to situations of daily life, including loss of attention, rumination, rigidity, moodiness [sad, anxious, and irritable], automaticity, and prejudice). Also, heightened self-awareness for positive changes was noted (eg, self-regulation toward calm, alert, self-accepting, prosocial, or assertive reactions).

With the whole happening of LAKA ... [patient explains] ... which makes you approach and do things less rigid and or short-sighted.

Patient #5

#### Serious Gaming Mechanisms That Lead to Learning Results (Mechanism and Outcome)

Intervention group patients who elucidated outcomes also spontaneously recognized active intervention elements (ie, resources). They spoke about *encounters*, *reflections*, mental training instructions, and debriefing. These were mostly connected to a form of *reasoning* that was commonly described as being *confronted with oneself*. Explanations of this common experience included awareness of alternate response options, emotional consequences of those responses, nonautomaticity, effort to maintain focus of attention, and transfers of gaming experiences into situations of daily life, for example:

You are confronted actually ... that is where awareness begins. In everyday life you often have those situations in which you do not even realize that you can go left or right. And yes, with LAKA you really get that choice and then you really have to start thinking.

Patient #2

Particular qualities of experience during gaming, including positive affect and *involvement*, were more often described by patients who also described positive learning results. However, none of the patients attributed their learning results to these gaming experiences explicitly and spontaneously:

I was really into it, the journey across the world ... and you can completely forget the world around you ... Where does that help for? Maybe that when you're busy with something ... you're just really focused on doing it, and not being distracted ... that concentration.

Patient #1

Patients’ reasoning seemed to correspond with care providers’ expectations, before the experiment (ie, second focus group), that LAKA might stimulate self-reflection and behavioral adaptation by showing opportunity thereof. After the experiment, care providers (ie, third focus group) emphasized the necessity of debriefing for many patients, for transferring their experiences into desired learning results. Debriefing group discussions provided the opportunity for patients to express their experiences *in* LAKA or *about* LAKA. These are vicarious experiences, mediated by the avatar, versus the nonmediated ones, such as declarations about a lack of identification with the avatar or the liking or disliking of entertainment-oriented features. Such nonmediated experiences were expected in the first focus group, when participants critically and jokingly wondered why one would not use something else to trigger experiences as a basis for discussion (eg, offering pastry). After the experiment, care providers agreed that the best results are gained by discussing both the mediated and nonmediated experiences in debriefing, albeit depending on situational and patient needs:

Those mini-games in between, which ought to be less valuable: for reflection, you can get more out of that than from the encounters, because: I never go to Istanbul, and I do not like temples. So, I just clicked something. That mini-game is really stupid, I had to start all over again! You can reflect nicely on that ... Why were you not interested in Istanbul? What does that say about you, your daily life, and your symptoms?

Health care provider

#### Characteristics of Intervention in Context for Triggering Mechanisms

Patients made both positive and negative remarks about serious gaming as part of the treatment program. They agreed that learning mechanisms were compatible to other educational and psychotherapeutic approaches in the rehabilitation program. Two patients explained that the opportunity for experiential learning supported the learning transfer. This offers a relative advantage to regular, more text-based modalities. However, outcomes were regarded as suboptimal with regard to the ambiguous and noncompelling feedback. Other generally experienced barriers were the limited duration, design quality aspects, and personalization or inflexibility of LAKA, as follows: (1) response options are prescripted, (2) the virtual world and the activities in it are neither exhaustive nor adjusted to with respect to what individual patients find important or valuable in their lives, (3) too much time could be spent on *nonactive* elements, and (4) a third-person, instead of first-person, perspective was used. Some example quotes follow:

It [reflection] should be, as far as I am concerned, more in the game and immediately after the choices you have made. Let consequences being “lived through” and then get back on; if this, then what?

Patient #7

The fact that all the answers that are given [options for responding in encounters] did not apply to me; I found that very difficult.

Patient #5

You are traveling the world, but it is actually about life. In that world you encounter something that you do not meet at home on the couch. In that journey you can put all kinds of aspects of life ... Do you bring your partner? Are you going alone? Those are actually very essential choices. Why does that man [avatar] have to travel alone?

Expert

The central planning of sessions by the clinic elicited varying views about the timing and presence of other patients in serious gaming sessions. One expert (first focus group) expected it to be better when sessions took place within a short period of time (ie, a maximum of 2 weeks). Patients clearly preferred that debriefings took place shortly after playing. This could matter for *confrontations* because recall of relevant serious gaming experiences gets more problematic over time. Some patients argued that when serious gaming was provided earlier, it would have helped more in combination with previous program elements. Others appreciated that gaming was not introduced earlier because its rationale would be more difficult to understand, or use of the computer-based modality would be considered more burdensome. One patient liked to have a debriefing with a care provider one-on-one, while other patients found it interesting to experience serious gaming (eg, performing meditation) in a room with other patients and to hear about the experiences of others.

In one scenario or one session, quite a lot happens: all the choices that people make. You could have concluded each session with an evaluation and reflect on what they just did. In the last session, it was not always clear what had happened and what they had experienced.

Health care provider #2

Before the experiment, care providers could only speculate about patient factors. They considered that processes for restoring social roles and pain acceptance throughout rehabilitation are too heterogeneous for making general predictive statements. After the experiment, both patients and care providers expected that a younger age, habit, or self-efficacy regarding technology usage contribute to positive patient expectations and experiences. Patients who expected benefit from serious gaming explained that they became aware of dysfunctional cognitions, moods, or behaviors. Respondents with low outcome expectations considered that they did not have emotional problems, or could control them, and focused on other means for handling their pain and other symptoms (eg, physical exercise) at that time. Care providers came to consider that patients who experience more difficulties and show a greater need for guidance when using LAKA (ie, to get in control and transfer experiences toward learning results) could be those who may benefit the most from it. This seemed consistent with what patients admitted about themselves, albeit in less explicit terms, for example:

When I first came there, and received such a tablet computer, games and such on computers really were not my thing. So, to me it was all “abracadabra” what happened. I have been fighting with myself for the first 50 minutes; what am I supposed to do with this? And then you try something.

Patient #5

To care providers, patient expressions of difficulties encountered when using LAKA were also useful input for debriefings. When guiding patients, care providers had prioritized reflection over convenience (eg, by offering practical information and assisting with forgotten passwords, computer and Internet settings, and game controls). A care provider explained how role performance in guidance could be influenced by an ambiguous attitude toward the compatibility of computer-mediated learning and personal work styles:

I think our role is to say at the beginning: “I am not going to tell you much. You get started, then I give information about how the device works, and eventually we will evaluate it” ... I must be self-critical: I was motivated to provide those sessions but skeptical, because games are not my thing. That is not good, because that influences your performance. Giving people little information has helped me not to be influenced.

Health care provider #1

### Adjudicating Formal Theory and Formulating Hypotheses (Step 2)

With regard to why serious gaming can enhance learning outcomes, *confrontation with oneself* was identified as a necessary form of reasoning. Based on this finding, SDT was chosen as an appropriate general theoretical framework for understanding underlying motivational and affective processes of learning from serious gaming by patients.

Various hypotheses were formulated and tested with available quantitative data for additional indications about the validity of ICMO elements. Hypothesis 1 focused on establishing a quantitative data pattern indicating the initially expected and qualitatively consistent premise that *mindfulness* is a rehabilitation mechanism, or interim learning outcome, that can be triggered by a serious gaming intervention. Hypothesis 2 was aimed at determining unique covariation between measures of learning results and potential gaming mechanisms, including valence of affect and sense of presence. Although it was initially considered possible to indicate mechanisms with a simple linear relationship between learning and valence of affect, there was no clear basis for this in qualitative results or in the SDT. A positive relationship between the subjective experience of involvement during play and mindfulness was hinted at in the qualitative data, but without explanation of why such a pattern would be generated. Hypothesis 3 focused on determining patient subgroups for whom particularly *good* or *bad* outcomes are attributable to serious gaming. Such an empirical pattern was seen as an indication that the self-discrepancies that some of the patients become aware of through serious gaming, that give reason to change behavior, may be absent in other patients. The latter being patients who find themselves free of unsolvable emotional stress, or assume that they can cope with their pain in different ways (ie, no current demand for mindfulness intervention). At the same time, individual inclinations to experience a lack of control could have a negative influence on the use of serious gaming and its results. Hypothesis 4 focused on remarkable covariation between perceived outcomes and factors of serious gaming intervention in context.

### Quantitative Analyses (Steps 3 and 4)

#### Overview

**Figure 2:**
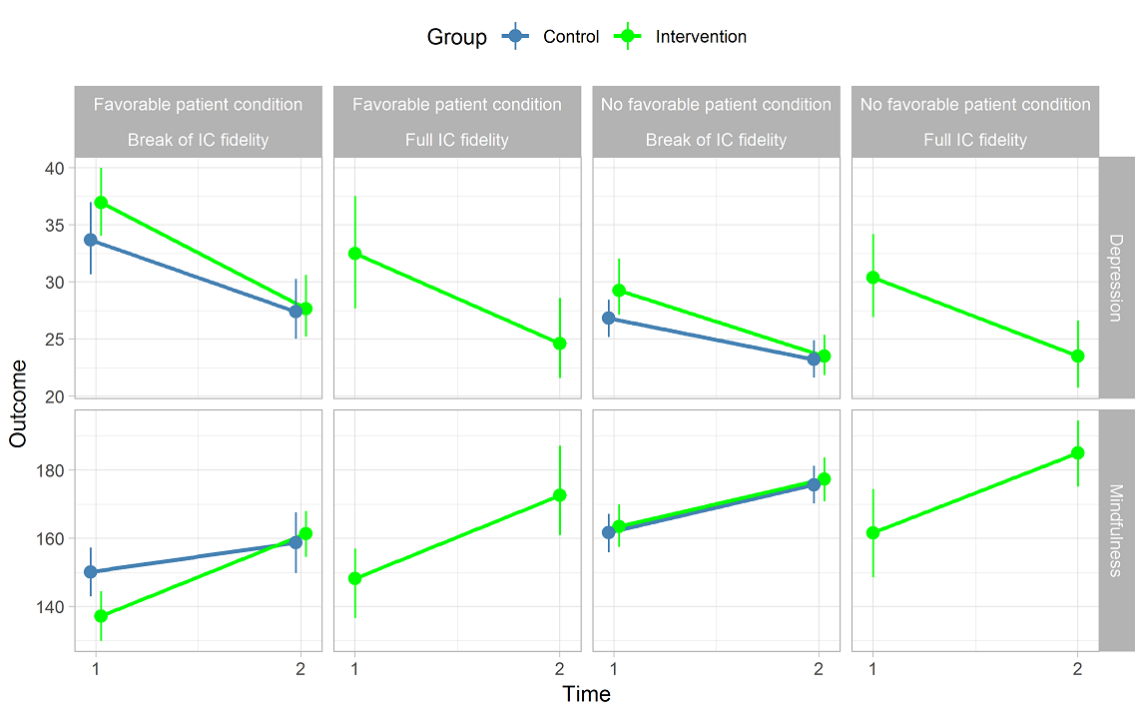
Outcome changes by conditions of the patient and intervention in context. Full intervention-in-context (IC) fidelity: debriefing is received in a group within 4 weeks after introduction and, at most, 2 days after completing at least 50% of a complete playthrough in LAKA. Favorable patient condition before gaming: at most, 45 years of age, high psychological symptoms, and active or pain coping levels are not high.

**Figure 3:**
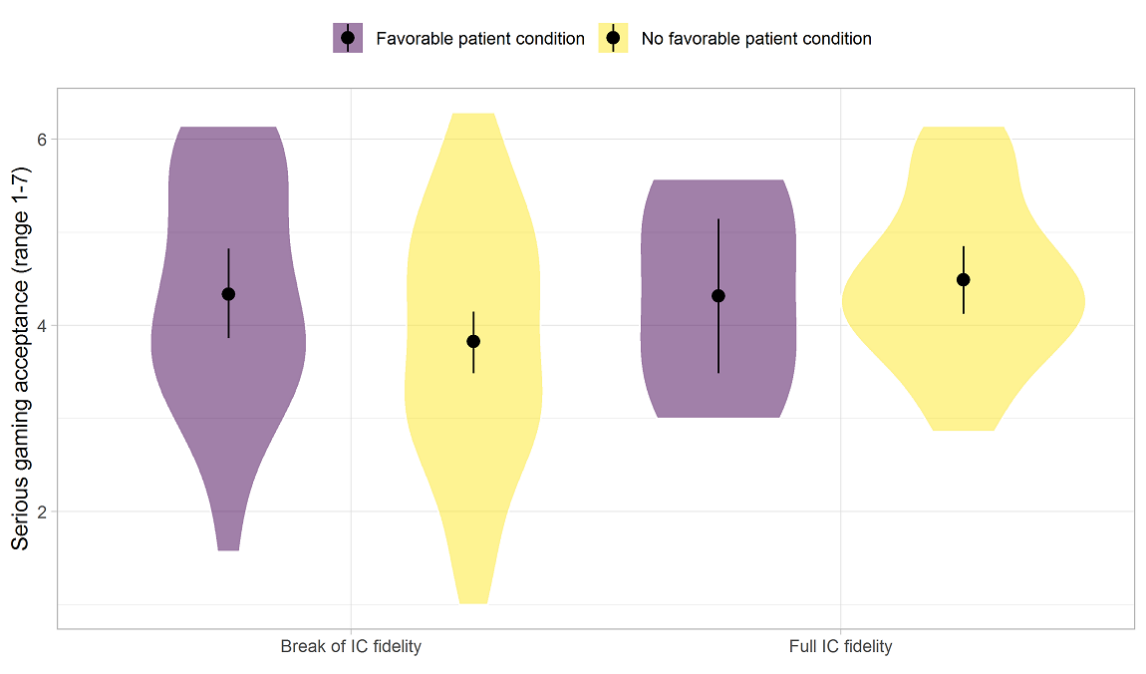
Gaming acceptance perceptions by conditions of the patient and intervention in context. Full intervention-in-context (IC) fidelity: debriefing is received in a group within 4 weeks after introduction and, at most, 2 days after completing at least 50% of a complete playthrough in LAKA. Favorable patient condition before gaming: at most, 45 years of age, high psychological symptoms, and active or pain coping levels are not high. The width of the "violins" represents the number of observations.

Figures 2 and 3 visualize scores for mindfulness change, depression change, and postgaming acceptance perceptions, separately, between contexts that were tentatively considered as *favorable* or *not favorable*. Relatively steep changes in mindfulness and depression scores in the *right* directions (see Figure 2) and more stable acceptance levels (see Figure 3) were visible for subgroups of patients of the intervention group with *favorable conditions* of the patient—at most, 45 years of age; *high* psychological symptoms; and not a high level of active coping or pain coping—or the intervention in context—debriefing is received in a group within 4 weeks after introduction and, at most, 2 days after completing at least 50% of a complete playthrough in LAKA.

#### The Effect of Group on Health Outcomes Mediated by Mindfulness (Hypothesis 1)

**Figure 4:**
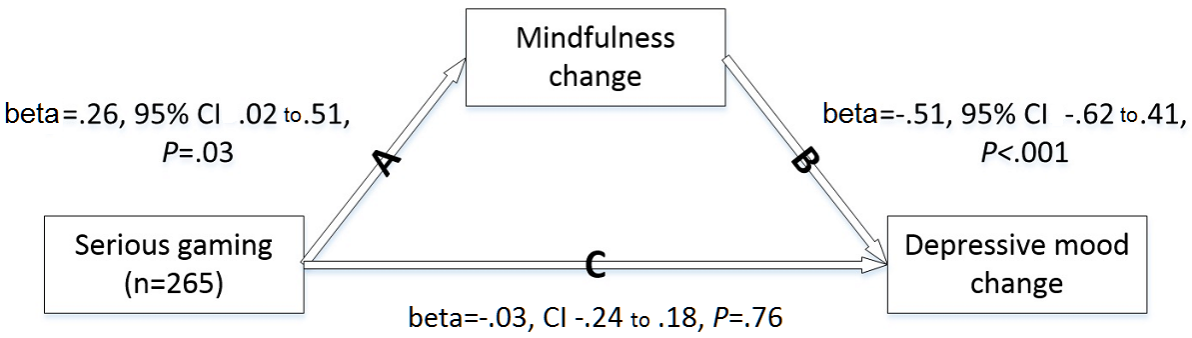
The mediated effect of serious gaming on depressive mood changes through mindfulness changes.

Figure 4 displays regression coefficients for the effects of study group on mindfulness change (path A), effects of mindfulness change on depressive mood change (path B), and effects of study group on depressive mood change after controlling for mindfulness change (path C). Together, the results show that mindfulness changes mediated the relationship between study group (ie, independent variable) and depression changes (ie, dependent variable). The indirect effect was very small (beta=-.14, 95% CI -.27 to -.02). Similar results were obtained with fatigue (beta=-.15, 95% CI -.29 to -.01) and current pain (0-100 numeric rating scale; beta=-.09, 95% CI -.19 to -.01) as the dependent variable.

#### The Relationships Between Serious Gaming Experiences, Learning, and Health Change (Hypothesis 2)

Mean levels for affect valence after serious gaming were 3.00 (SD 0.91, range 1-5) for positive affect and 1.53 (SD 0.63, range 1-5) for negative affect. Mean scores for the subscales of sense of presence were 2.59 (SD 1.29) for involvement and 2.40 (SD 1.05) for realism. Intercorrelations between positive affect, sense of presence, and game acceptance were of moderate sizes (.57<ρ<.59, *P*<.001). Regression of *mindfulness* change on positive affect, negative affect, involvement, realism, and outcome expectations (ie, control variable) only showed statistical significance for involvement (N=113, R^2^=.10, beta=.36, *P*=.001).

#### Differences in Intervention Effects on Outcomes by Patient Factor (Hypothesis 3)

Age appeared to be the only patient factor with a notable association with game acceptance scores (b=-.025, *P*=.04). Moderated mediation analysis did not show statistically significant differences in the indirect intervention effect on depression change through mindfulness change between the two age groups.

A stronger indirect intervention effect on health outcome change was seen in the subgroup of participants without *high* active coping scores. For absence (score<21) versus presence of high active coping (213/265, 80.4%), the moderated mediation index amounted to -.26 (95% CI -.06 to -.52).

**Figure 5:**
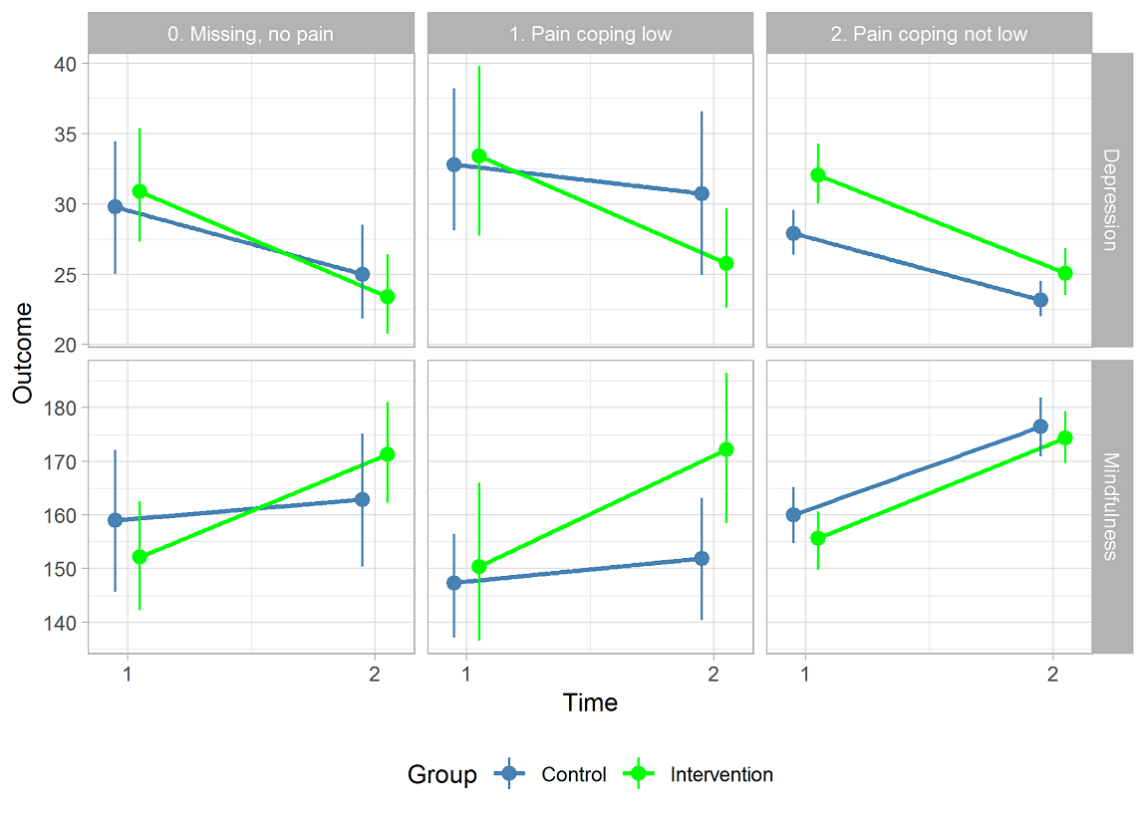
Group differences in average depression and mindfulness changes by pain coping.

Within the subgroup of 33 out of 214 patients (15.4%) with low pain coping scores (18/121, 14.9%, from the intervention group and 15/93, 16%, from the control group), the indirect effect estimate (beta) was -.79 (95% CI -1.50 to -.31) stronger than within the 181 out of 214 (84.6%) other patients. Figure 5 shows that changes of mindfulness (ie, positive) and depressive mood (ie, negative) were particularly weak for control group patients with low pain coping.

Finally, no difference in the overall indirect effect was seen between subgroups determined by high or very high psychological distress: index of moderated mediation was -.19 (95% CI -.40 to -.02).

#### Differences in Outcomes by Intervention in Context (Hypothesis 4)

Multilinear regression models showed that none of the objective indicators for intervention in context had a notable direct association with health outcome changes. For mindfulness change, model prediction improved by the addition of the dummy variable for *group* as a debriefing session structure (R^2^_change_=.03, b=.36, *P*=.04). Generally, addition of the planning variables did not improve the mindfulness change model (R^2^_change_ =.05, *P*=.073). The parameter estimates were as follows: b=-.001, SE=.006, *P*=.84 for the date differences between preintervention assessment (t1) and the introduction session; b=-.02, SE=.01, *P*=.06 for the date differences between introduction and debriefing; and b=-.02, SE=.01, *P*=.03 for date differences between debriefing and postintervention assessment (t2). Bootstrapping or removing outlying mindfulness change values (Z>3 or Z<-3) generally accentuated the directions in which these findings pointed. Regressing game acceptance perceptions on a dummy variable for debriefing *shortly* after playing LAKA for the last time (ie, within less than 3 days) also resulted in model improvement (R^2^_change_=.08, b=.72, SE=.23, *P*=.002).

### Mixing the Qualitative and Quantitative Results (Step 5)

Two original ICMO-Cs were constructed by updating initial formulations on the basis of the qualitative and quantitative results (see Textbox 4).

**Final configurations of contexts, mechanisms, and outcomes.**

Intervention-context-mechanism-outcome configuration (ICMO-C) 1. Serious gaming acceptance perceptions are a feasibility mechanism and a context for learning and health outcome change that get hindered when:

- design qualities with respect to clear feedback and tailoring to recipient preferences are limited (I in C);
- players attribute negative perceptions to their older age (C); and
- implementation processes (ie, central planning) do not facilitate immediate debriefing after play for sharing available memories (I in C).

ICMO-C 2: A stronger self-awareness in daily life (ie, rehabilitation mechanism or serious gaming outcome) and subsequent changes in health effects (ie, rehabilitation outcomes) are triggered by involvement in serious gaming tasks, wherein both the actual self and discrepant self-scripts (ie, serious gaming mechanisms) are being processed. This occurs when:

- the innovation of treatment delivery modes is relatively advantageous (ie, adds experiential learning) and compatible with the rationale of a larger treatment program (I in C);
- the patient has limited inclination to manage with stress or pain in active or variegated ways (patient in C); and
- organizational implementation processes are characterized by adequate planning of patient guidance; sessions can address an actual need or discrepancy in a timely manner and debriefing is followed in a small group with other patients (I in C).

## Discussion

### Summary of Findings and Comparison With Existing Literature

#### Overview

**Figure 6:**
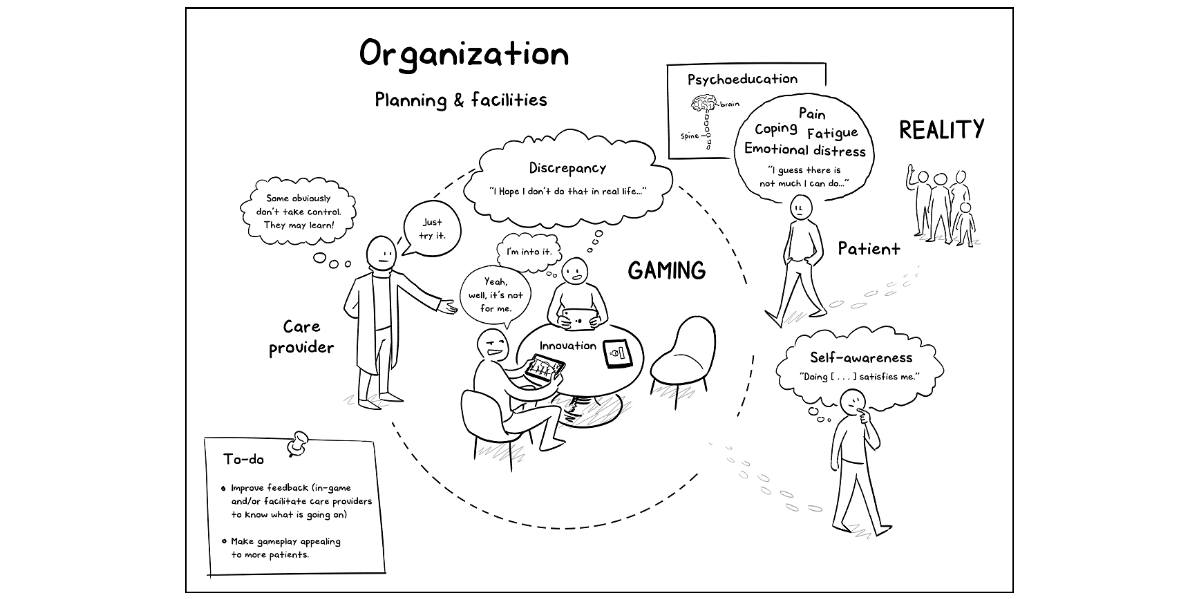
A visual summary of program theory about serious gaming during the multidisciplinary rehabilitation of patients with complex chronic pain or functional somatic syndrome.

This study informs future recipients, developers, evaluators, implementers, and policy makers about using, developing, or implementing serious gaming in a health care context. The question was how, why, for whom, and when facilitating additional serious gaming during multidisciplinary rehabilitation is effective for patients with CP or FSS. Qualitative and quantitative research was used to update an initial theory about mechanisms through which elements of a serious gaming intervention—with the game LAKA—lead to relevant positive effects of learning and physical and emotional symptom reduction. Moreover, insights have been developed as to which patients and circumstances of intervention such effects do and do not occur. Figure 6 presents a visual representation of the identified processes (ie, corresponding to Textbox 3). In the following sections, the contributions of this study to the further development of initial program theory are summarized (see Textbox 2).

#### How Serious Gaming Facilitates Outcome Change During Multidisciplinary Rehabilitation

The *how* question was addressed by (1) asking patients how serious gaming during multidisciplinary rehabilitation influenced health outcomes, if perceived to be the case, and by (2) estimating how much additional change in learning about mindfulness and health outcomes was attributable to the serious gaming intervention and subjective experiences thereof. First, the findings supported the premise that serious gaming can be a relevant assistive tool to activate a rehabilitation mechanism of open and nonjudgmental *self-awareness*. Second, initial ideas about how serious gaming interventions can facilitate learning in this respect can be refined based on our findings. Prompts that encourage patients to reflect on discrepancies between their current behavior and their goals constitute a *resource* within the LAKA game. It evoked a kind of *reasoning* that patients referred to as a *confrontation with yourself*. In addition, the initial beliefs that debriefings facilitate learning transfers between participants and contexts were supported [52,59]. An unexpected finding was that a lack of perceived behavioral control in patients during serious gaming sessions, according to care providers, signaled a need for support as well as a potential to benefit from serious gaming. Third, this study refuted a general and simple positive linear pattern of stronger learning outcomes as patients experience more positive affect or virtual presence while playing a serious game. It was found, however, that a stronger subjective involvement (ie, attention devoted during serious gaming) had a positive association with mindfulness and symptom changes. The explanation for this remained implicit.

#### Why Serious Gaming Can Facilitate Learning and Subsequent Health Changes

The *why* question was addressed by adjudicating theory that can serve as a general *underlying* explanation for the *being confronted* experiences of patients. The SDT was chosen as a good starting point for predicting how mindfulness-based serious gaming can support learning and related affective and motivational processes. SDT is considered complementary to a model of mindfulness for explaining mechanisms [37]. Mindfulness can be seen as a way of processing self-discrepancies through which one is capable of moderating their affective and behavioral consequences [37,92]. This is illustrated by the common instructions of focused-attention meditation exercises that were also instructed in LAKA: *gently* return focus of attention to a chosen mental or sensory object (ie, *ideal*) when the present object of attention (ie, *actual*) is discrepant from that object. From this, it follows that *self-awareness* as a learning result from serious gaming concerns both the actual behavior of patients in daily social life as well as new or existing *noncontingent* scripts (ie, behavioral ideals and norms).

#### For Whom Does Serious Gaming Facilitate Outcome Change

Initially there were hardly any starting points for developing propositions regarding which patients with CP or FSS serious game intervention would be more or less effective. Findings from this study suggested theoretical extension with patient factors for feasibility and effectiveness. First, it is now suggested that perceptions of serious gaming acceptance can be weaker in patients with a relatively older age. This does not say that age, per se, is an explanation. Second, chances of a relevant effect of serious gaming on depressive symptoms may be greater in patients who experience less control over the consequences of stressors or pain in their daily lives. In an identifiable subgroup of patients with lower scores on active coping and pain coping scales, the average intervention effects were *small* or *medium* instead of *very small*, as found for all patients together [30]. This finding is remarkable as more active coping predicted better health outcome changes during regular rehabilitation. Moreover, pilot study and qualitative data suggested that a more active coping style facilitates usage of serious gaming. On the other hand, the effectiveness of serious gaming can now be called into question for patients who do have a tendency toward active and variegated ways of coping with stress or pain, and who already improve when receiving other means of treatment.

#### When Serious Gaming Affects Outcome Change

Circumstances determining the generation of mechanisms that lead, or do not lead, to serious gaming outcomes were examined qualitatively by asking patients for strengths and weaknesses of a serious gaming intervention during multidisciplinary rehabilitation. Responses were classified by factors on innovation, care provider, and organizational levels. Where available, quantitative data were used to identify patterns of covariation between indicators of relevant circumstances of serious game intervention in context and acceptance perceptions, learning outcomes, or health outcomes.

Based on the findings, it is proposed that blended serious gaming interventions trigger the mechanisms of effectiveness (1) when adding a relatively advantageous feature to the treatment (ie, experiential learning in addition to text-based psychoeducation), (2) when these features are compatible with the rationale of existing treatment (ie, adaptation to a CP or FSS condition by shifting attention away from self-scripts that actually depend on a known or indefinite underlying pathology toward scripts that are not), and (3) when organizational processes of planning and facilitation of instruction and feedback sessions are being implemented at high fidelity (ie, central planning and facilitating of debriefing in small patient groups). Moreover, serious gaming interventions may be perceived more positively by patients (1) when debriefing is always provided immediately after play or automatic feedback functions are adequate and (2) when software design adapts well to recipient needs and preferences (eg, regarding game environments and freedom of choice).

### Strengths and Limitations

#### Paradigm

A major strength of this study is that the realist evaluation approach led to transferable findings on a theoretical level. A new example is added to a few previous ones about how quantitative methods can be used within a realistic evaluation paradigm [93-95]. Limitations related to the use of *realistic* principles are due to the late adoption during evaluation. This could have influenced initial program theory formulations (eg, conflation of intervention characteristics with mechanisms in the protocol stage) and, therefore, possibly also the data collection [96]. Perhaps more elaborate initial theoretical development could have enabled the study to (1) pose more or better follow-up questions to participants during interviews, (2) narrow the sets of sensitizing concepts and topics, or (3) determine more specific criteria for data saturation. Also, it could have led to different procedural choices. For example, interview data that resulted from the procedure of matching control group with intervention group cases failed to result in similar cases, with the exception of exposure to serious gaming and outcomes thereof. This was considered a reminder that a counterfactual logic of analysis does not suit a realist approach of discovering generative causal intervention effects.

#### Design and Procedures

General strengths and weaknesses of the mixed methods design were previously reported [60,96], but additional points on execution are noted here. Embedded experimental mixed methods ideally suited the questions and realistic approach of this study [97]. To realists, various research methods are commensurable and complementary and none is generally preferable. Controlled experiments can provide precise estimates of outcome pattern attributions to experimental conditions, but different methods are needed when well-defined ideas are lacking about how and why intervention effects are generated [98].

The validity of our findings varies across the study objectives and corresponding program theory elements. This is because not all qualitative findings were also triangulated with quantitative indications. As the study protocol had to be ethically approved before data collection started, the selection of quantitative measures could not be based on tentative ICMO-Cs after qualitative investigation. In realist evaluation, like in any other methodological approach, data collection is ideally informed by the most recent theoretical insights. Moreover, intervention and context factors had limited variation because only a single setting and a single serious game were studied. However, strong representativeness was achieved in the sampling of patients.

#### Methods

Finally, the findings of this study are to be interpreted in the light of theory development. Future studies are needed to proceed in the development of realistic propositions with newly collected data and complementary methodological strengths. With regard to the qualitative methods of this study, trustworthiness is supported by some techniques (ie, part of the qualitative data was independently coded). Regarding our quantitative analyses, limiting the number of variables by formulating hypotheses before quantitative data inspection, using qualitative findings, reduced the risk of capitalizing on chance for finding statistically significant results. Moreover, calculations of key parameters were repeated after particular methodological changes in order to check for the robustness of our findings. More detailed considerations about sensitivity analyses, data quantity, data quality, and missing data are presented in Textbox 5. Still, many decisions related to the calculations of variables and model specifications were not fully detailed in the study protocol or trial register.

**Additional details on the quantitative analyses.**

- Sample size calculation did not specifically anticipate the testing of complicated, configurational propositions. Still, the 265 out of 275 cases with complete outcome data provided sufficient statistical power for moderated mediation modeling unless true direct effects were very small [98].
- Potentially relevant influences on the results due to nonresponse to the postgaming survey were explored by running chi-square or Student t tests on the differences in patient factors and gameplay behaviors between those who did and did not complete this survey.
- We did not apply methods to correct for multiple hypotheses testing.
- The psychometric qualities of the mindfulness measurements available for this study were assessed during an audit by an independent knowledge institute, but not during the peer-review process of an international scholarly journal.
- The results of sensitivity analyses showed that none of the presented estimates changed in an important manner after methodological changes. First, an intention-to-treat analysis was performed that yields unbiased estimates of the group × time effect on the intermediate outcome (ie, mindfulness), assuming that the outcome data of some respondents (ie, <4%) were missing at random. The moderated mediation models were refitted after replacing health outcome and learning outcome indicators with different residual posttreatment scores (t2): that is, the residual scores after regression on both the pretreatment score (t1) and the possible confounding variables. The latter refers to the variables for which a potential difference was found between the study group averages [60]. Finally, sensitivity of the coefficients produced with multivariable linear regression models was checked by the performance of bootstrapping with 1000 samples and/or removal of outlying cases on the dependent variable: standardized scores >3 or <-3.

### Future Directions

This study illustrates that relevant insights for optimizing biopsychosocial management of CP or FSS (ie, including eHealth) can be developed with an explanatory and context-sensitive evaluative approach. Ideally, such research precedes and goes along with design and assessments of cost-effectiveness and quality improvement with suitable experimental methods (eg, randomized controlled or pragmatic, single- or multicenter, and individual allocation or stepped-wedge methods) [73]. Previously, researchers made similar suggestions for researching effectiveness of treatments for patients with CP, FSS, or mental health problems [31,45,99-101]. After all, consensus building about appropriate allocation and tailoring of treatment to patients has been relying on limited evidence [102]. As realist approaches focus on such issues, there is a large but yet unproven promise for methodological progress to support new insights for intervention quality improvement, treatment allocation decisions, personalization, and setting patient expectations. Specific areas for future theory-oriented research on serious games are implied in Table 4. This table gives an overview of considerations about how the findings of this study relate to those of other studies in various disciplines [1,3,30,31,52,53,59,68,85,87,96,103-116].

**Table 4:** Previous research findings related to the results of this study.

Question	Comparable research findings
How or why (rehabilitation mechanism)	The mediation analysis in this study (see Figure 4) showed similar results to a previous meta-analytic mediation analysis, which was mostly based on experimental comparisons of mental health outcome data with intervention groups that received comprehensive mindfulness programs and *passive* control groups (ie, standard treatment or waiting-list controls) [103]. Both studies found a *very small* indirect average effect of mindfulness-based intervention on mental health outcomes via *small* effects on mindfulness changes. The meta-analysis, however, found a significant direct intervention effect that was not seen in this study. A plausible explanation for this difference is that other ways in which mindfulness interventions work are nonspecific and had already been elicited by the other face-to-face-delivered techniques (eg, neuroscience education and cognitive restructuring) that the control group patients in our study received. In line with previous studies that suggested a limited sustainability of behavioral change effects, this study showed a decrease in the learning results of participants in the intervention group as the amount of time between debriefing and posttreatment assessment increased [1].
How or why (serious gaming mechanism)	Drawing attention to discrepancies between a person’s current behavior and the person’s previously set outcome goals, behavioral goals, or action plans constitutes an acknowledged behavioral change technique [104]. This technique apparently was the strongest, or most distinctive, trigger among other techniques that were integrated in the game LAKA [96]. LAKA does not contain explicit educational elements to change specific cognitive antecedents of health behaviors, as has been common in other health behavior games [52]. A crucial property for influencing self- or emotional regulation processes with games is user identification with avatars in storylines [105]. Herein, avatars have heroic or antiheroic qualities and challenges (eg, growing and defeating an antagonist). In LAKA, the avatar starts his or her journey in a state of discrepancy with respect to a relevant goal or ideal, and is committed to change. Users are then given control over avatar behaviors that determine the goal state. Distraction could be an alternative reason for a positive association between involvement in serious gaming and changes in pain intensity [53]. Finally, findings of this study correspond with a previous study that discovered positive behavioral change effects by eliciting self-discrepancy in videogames when users are primed to self-regulate with a prevention instead of a promotion focus [106]. Compared to other virtual environments and games, the average level of involvement in LAKA had a similar, relatively high, level [85]. Previous studies suggested that behavioral change outcomes are positively moderated by involvement in immersive virtual environments, as compared to, for example, text-based webpages [52,107-109].
For whom	Associations between age and technology acceptance have been inconsistent across contexts of use [110]. Previous pilot results hinted more specifically at an explanation for a pattern of lower acceptance with higher age. There is a positive moderation effect of age on the relationship between anxiety about technology use and acceptance [68]. Explanations may lie in general beliefs that games require young people’s skills, or that older people are usually late adopters of technology. Previous work supports the finding that both *active* coping and pain coping, using many different strategies, are often, but not always, adaptive for patients with chronic pain as they are often used in the flexible coping by patients who remain hopeful and optimistic about being able to achieve important life goals [3]. Improvement of mindfulness, through acceptance of aversive experience, emotional regulation, and perceived support, can enhance adaptation to chronic pain (eg, moderate depressive symptoms) independently of pain coping [111,112]. Furthermore, psychological approaches may generally be more effective in patients who have more to gain with respect to emotional functioning [30,31].
When	The importance of adopting a valid rationale in developing games for health was emphasized in existing guidelines [113]. The indications from this study that immediate face-to-face interactions are important correspond to the notion that debriefings are necessary for generally satisfying learning experiences after serious gaming [59]. The role of group dynamics for effective debriefings were previously touched upon, but not well understood [59]. A previous implementation study of Internet-based cognitive behavioral therapy identified intervention adaptability as an important innovation-level barrier for usage [114]. Care providers’ initial skepticism about serious gaming corresponds to previous findings on care provider perspectives on digitalized interventions, including serious gaming, for mental health improvement [115,116]. The proportions of patients who logged in to use LAKA (n=155), among those who were part of the intervention group (n=156) or had access (n=171), were much higher under the circumstances of this evaluation study than in the previous feasibility pilot (ie, n=71, n=116, and n=410, respectively). During the pilot, serious gaming was available only for one computer platform. It was also not an embedded treatment component and did not include care provider support [68]. Under such *noncommittal* circumstances, usage may be less well explained by a process of *going with the flow*, and more so by individual planning processes [87].

### Conclusions and Recommendations

Following a realistic approach, this study started with the selection of potentially relevant theories and ended with an integrated fallible model [98]. Serious gaming (ie, a 4-hour intervention during an intensive multidisciplinary rehabilitation program) can offer relevant additional opportunities for enhancing self-awareness and reducing emotional symptoms by triggering increased attention and self-discrepancies. This can be achieved in CP or FSS patients who experience relatively less control over their current stress or pain. This also depends on the fidelity of implementation processes (ie, planning and facilitating) in such a way that gaming can be immediately followed up by exchanging experiences between patients under professional guidance, toward goals compatible with the overall treatment. At the same time, there is no sign as of yet of potential clinically relevant effects of serious game interventions in other treatment circumstances and among patients with CP or FSS.

This already offers some transferable insights for patients, care providers, health care organizations, insurers, policy makers, and businesses who will make future decisions on the future implementation of serious gaming. It may not be wise to offer this form of serious gaming, with similar late timing during rehabilitation, to all patients, including those claiming that their stress and pain are already under control.

Realizing the promise of serious gaming depends on ongoing efforts to develop transferable understandings of how they work, for whom, when, and why. In doing so, focus can be informed by perspectives of important stakeholders. The theoretical scope could be extended to the outcome domains of quality of life of patients, adoption and implementation by organizations, and cost-effectiveness. Understanding about the mechanisms of learning from serious games could be improved by applying SDT when formulating predictions about cognitive and emotional consequences of certain changes in the design and context of use. Finally, future studies could zoom in and out on micro- or macro-level conditions for serious gaming with impact. For example, patients, software developers, and data scientists could collaborate to optimize software (eg, in-game feedback) for adaptation to recipient characteristics for learning and health improvement. Care providers can also be involved in optimizing feedback provision (eg, enabling them to interpret log data) and in optimizing the fit with work routines. Lastly, on an organizational or societal level, policy makers could participate in theorizing on preconditions for implementation, such as financial arrangements and dissemination platforms.

## Appendix

Multimedia Appendix 1 Initial developer assumptions and evaluators’ interpretations on the basis of relevant existing scientific theories.

Multimedia Appendix 2 Additional information about the mindfulness measure.

Multimedia Appendix 3 Coding illustrations for individual-level intervention-context-mechanism-outcome configurations and barriers.

## Abbreviations

CP: chronic pain
FSS: functional somatic syndrome
ICMO: intervention-context-mechanism-outcome
ICMO-C: intervention-context-mechanism-outcome configuration
ICT: information and communication technology
SDT: self-discrepancy theory
t0: baseline
t1: intermediate time point after 8 weeks of treatment
t2: posttreatment after 16 weeks
