# Supplementary material for: Serious Gaming During Multidisciplinary Rehabilitation for Patients With Chronic Pain or Fatigue Symptoms: Mixed Methods Design of a Realist Process Evaluation
Source: J Med Internet Res. 2020 Mar 9;22(3):e14766. doi: 10.2196/14766 (PMC7091046; doi:10.2196/14766)
Supplement: Multimedia Appendix 1 [file jmir_v22i3e14766_app1.pdf]

***Multi-media appendix 1: initial developer assumptions and evaluators' interpretations on the basis of relevant existing scientific theories***

| I-C-M-O component | Developer assumptions | Related theory (interpretation) |
|---|---|---|
| Intervention | The game is of a relatively short duration, and is also not intended to attract players for an extended period of time. Rather it promotes continued practice of instructed skill by any means outside the game (in the 'real' world). | Potentially important qualities of serious gaming, as an innovation of multidisciplinary rehabilitation, were inferred from two comprehensive frameworks [1, 2]. Some of the characteristics included in both frameworks include relative advantage ("stakeholders' perception of the advantage of implementing the intervention versus an alternative solution"), adaptability/flexibility/personalization ("the degree to which an intervention can be adapted, tailored, refined, or reinvented to meet local needs", complexity ("perceived difficulty of the intervention, reflected by duration, scope, radicalness, disruptiveness, centrality, and intricacy and numbers of steps required to implement"), and design quality ("perceived excellence in how the intervention is bundled, presented, and assembled"). Relative advantage could be described in terms of strategies for behavioral change that, within a context of treatment, is only provided by means of serious gaming. |
| Context | The serious gaming intervention was developed with the local circumstances of multidisciplinary rehabilitation in mind, and expectations regarding the context in which serious gaming would or would not be feasible were shaped after a pilot study (see right column).<br>Within rehabilitation, serious gaming sessions are planned after psycho-educational components (including stress management, cognitive restructuring, and meditation) to enable complementary engagement in - and transfer of - learning. | The (fixed) context of implementing the serious gaming intervention in the rehabilitation program for the duration of the evaluation study could also be characterized by comprehensive models of potential implementation context factors [1]. From this perspective, there may have been a particularly favorable climate and culture for implementation within the clinic, as professionals were used to expectations about being assigned to perform their role in developing the treatment program. There was no particular urge to implement. Funding to sustain serious gaming as a treatment component had not been organized. Professional had not taken part in the previous feasibility pilot from which all key specifications of the serious gaming intervention were established (i.e. with opportunity to play LAKA on site and integration as a normal part of treatment) [3], but some degree of participation was enabled for taking more detailed decisions regarding the local implementation activities. Variation in factors related to the care providers was therefore not excluded. Another model considered to interpret context factors that may arise throughout the study was the consolidated model for implementation science, which was developed to extend coherence between theories of implementation contexts of health services [2]. Both models include an extensive set of factors of which only some may play an important role for a particular health service. |
| Mechanisms | Rehabilitation activities were aimed at improving an individual property labelled as 'mobility of mind': flexibility in accommodating the dynamic processes of: 1) actually | This learning content may converge with and diverge from related concepts known in published scientific literature, including:<br>- Coping flexibility under conditions of chronic pain [5]. The game challenges vicarious responding in a way that reflects acceptance of adversity in the |

| | | |
|---|---|---|
| | participating in private, social, and work roles, and 2) sustaining/developing the ability to participate. Ability to participate depends on four interdependent aspects: (1) symptoms (signals of organ system injury or disturbance in shaping and controlling one's life or 'existence'), (2) autonomy, (3) perspective ('to see one's opportunities for finding meaning in life…'), and (4) valuing generosity, moral discipline, patience, enthusiastic perseverance, and mental stability. This set of properties regarding individual functioning is assumed to reflect changes in the central nervous system.<br><br>Experts (educationalists) brought in ideas in early design stages about distinctive ways and reasons why serious gaming could work that were built on experiential learning theory [4]. This emphasizes the role the whole 'experience', including affect rather than (specific forms of) 'cognition' only, in the learning process. Accordingly, initiators claimed a tacit understanding that some patients would learn better, more easily, or faster, through gaming experiences. | context of daily life-like social interactions and engage in adaptive behaviors.<br>- Eudaemonist process of psychological well-being [6]. The storyline of the game intends to challenge experimenting with behavioral choices that align with a health promoting way of living. That is: rich in purpose and meaning (act in accordance with a set of values/principles), continued growth (overcome adversity), and quality ties to others (respond well with others).<br>- Autonomously motivated pro-social behavior [7]. The design of the game provides opportunity to vicariously display prosocial actions. This may result in an (vicarious) state of satisfaction if the quality of the motivation of the chosen act is 'autonomous'.<br>- (Neuro) psychological processes associated with mindfulness oriented practices: self-awareness, self-regulation, and self- transcendence [8]. The game tasks demand that a player: (a) is self-aware (that one is represented by an avatar), (b) regulates actions (for the avatar/self), (c) selects prosocial acts (transcends immediate personal interests), and (d) follows instructions for focused attention and open monitoring exercises (meditate).<br><br>Plausible explanations of why video game mechanics may affect the outcomes in a distinctive way:<br>- Generally, distinctive features of serious gaming, including interactivity and debriefings in support of experiential learning, could strengthen (moderate) effects of behavioral change content on outcomes [9]<br>- Social cognitive theory: enhancing self-efficacy by embedding vicarious learning techniques in health behavior games [9].<br>- Self-determination theory: gaming provides opportunity to vicariously represent valued self-identities (be in an environment that provides autonomy, competence, and relatedness) [10, 11].<br>- Meta-cognitive processing is a likely consequence of, and characterizes interpersonal interactions in the context of video game-play [12].<br>- Tasks within the game 'LAKA' could be interpreted from the behavioral change taxonomy version 1 [13]. In this regard, serious gaming provides vicarious learning opportunities that are not present in other (computer-based) interventions for the target group [14]. |
| Outcomes | The rehabilitation program was characterized as having a ('four dimensional') bio-psycho-social-spiritual/existential treatment approach: particularly emphasizing on improving social role participation and psychological | - Both in practice and research outcomes of (multidisciplinary) rehabilitation are commonly classified in accordance with Categories of the International Classification of Functioning (ICF).<br>- Herein, outcomes of physical and emotional functioning are commonly seen as relevant in populations of patients with chronic pain [16, 17].<br>- ICF categories that could be specifically affected by |

| | | |
|---|---|---|
| | well-being [15].<br><br>Although many rehabilitation activities are not directly targeted at alleviating physical symptoms, they remain to be considered important as being cause for seeking care. Most of the patients treated in the clinic also experienced fatigue complaints and part of the patients are chronically fatigued but not in pain, characteristics of fatigue are also considered to be an important outcome dimension.<br><br>Outcomes are assumed to improve in conjunction, mostly due to changes in a single coherent set of mechanisms (‘mobility of mind’). | serious gaming are (higher) mental and emotional functions, and activities and participation domains 6-9. Therefore, emotional functioning was regarded as a plausible and relevant outcome.<br>- Pain (interference), mood disturbances, and fatigue are highly interrelated [18, 19].<br>- Sustainable small to medium sized effects on physical and emotional functioning and quality of life outcomes were found in a meta-analysis of the effects of mindfulness or acceptance oriented strategies in patients with chronic pain [20]<br>- Expectable intermediate outcomes for the approach taken by the serious game may converge with and diverge from similar plausible targets (mediators of effectiveness) in interventions with similar approaches targeted at patients with chronic pain, such as experiential avoidance (-) vs. psychological flexibility (+), mindfulness (+), rumination (-), catastrophizing (-) [21, 22]. There is however, a lack of understanding about which mechanisms are particularly affected by what strategies. |

## References


1. Grol R, Wensing M. What drives change? Barriers to and incentives for achieving evidence-based practice. Medical Journal of Australia. 2004;180(6):S57.
2. Damschroder LJ, Aron DC, Keith RE, Kirsh SR, Alexander JA, Lowery JC. Fostering implementation of health services research findings into practice: a consolidated framework for advancing implementation science. Implementation science. 2009;4(1):1.
3. Vugts MA, Joosen MC, van Bergen AH, Vrijhoef HJ. Feasibility of Applied Gaming During Interdisciplinary Rehabilitation for Patients With Complex Chronic Pain and Fatigue Complaints: A Mixed-Methods Study. JMIR serious games. 2016;4(1).
4. Kolb DA, Boyatzis RE, Mainemelis C. Experiential learning theory: Previous research and new directions. Perspectives on thinking, learning, and cognitive styles. 2001;1(8):227-47.
5. Hayes SC. Acceptance and commitment therapy, relational frame theory, and the third wave of behavioral and cognitive therapies. Behavior therapy. 2004;35(4):639-65.
6. Ryff CD, Singer BH. Know thyself and become what you are: A eudaimonic approach to psychological well-being. Journal of happiness studies. 2008;9(1):13-39.
7. Weinstein N, Ryan RM. When helping helps: autonomous motivation for prosocial behavior and its influence on well-being for the helper and recipient. Journal of personality and social psychology. 2010;98(2):222.
8. Vago DR, David SA. Self-awareness, self-regulation, and self-transcendence (S-ART): a framework for understanding the neurobiological mechanisms of mindfulness. Frontiers in human neuroscience. 2012;6:296.
9. Baranowski T, Buday R, Thompson DI, Baranowski J. Playing for real: video games and stories for health-related behavior change. American journal of preventive medicine. 2008;34(1):74-82. e10.
10. Ryan RM, Rigby CS, Przybylski A. The motivational pull of video games: A self-determination theory approach. Motivation and emotion. 2006;30(4):344-60.
11. Przybylski AK, Weinstein N, Murayama K, Lynch MF, Ryan RM. The ideal self at play: The appeal of video games that let you be all you can be. Psychological science. 2012;23(1):69-76.
12. Kim B, Park H, Baek Y. Not just fun, but serious strategies: Using meta-cognitive strategies in game-based learning. Computers & Education. 2009;52(4):800-10.
13. Michie S, Richardson M, Johnston M, Abraham C, Francis J, Hardeman W, et al. The behavior change technique taxonomy (v1) of 93 hierarchically clustered techniques: building an international

consensus for the reporting of behavior change interventions. Annals of behavioral medicine. 2013;46(1):81-95.
14. Vugts MA, Joosen MC, van der Geer JE, Zedlitz AM, Vrijhoef HJ. The effectiveness of various computer-based interventions for patients with chronic pain or functional somatic syndromes: A systematic review and meta-analysis. Plos One. 2018;13(5):e0196467.
15. Garschagen A, Steegers MA, Bergen AH, Jochijms JA, Skrabanja TL, Vrijhoef HJ, et al. Is there a need for including spiritual care in interdisciplinary rehabilitation of chronic pain patients? Investigating an innovative strategy. Pain Practice. 2015;15(7):671-87.
16. Dworkin RH, Turk DC, Wyrwich KW, Beaton D, Cleeland CS, Farrar JT, et al. Interpreting the clinical importance of treatment outcomes in chronic pain clinical trials: IMMPACT recommendations. The Journal of Pain. 2008;9(2):105-21.
17. Schwarzkopf S, Ewert T, Dreinhöfer K, Cieza A, Stucki G. Towards an ICF Core Set for chronic musculoskeletal conditions: commonalities across ICF Core Sets for osteoarthritis, rheumatoid arthritis, osteoporosis, low back pain and chronic widespread pain. Clinical rheumatology. 2008;27(11):1355-61.
18. de Heer EW, Gerrits MM, Beekman AT, Dekker J, van Marwijk HW, de Waal MW, et al. The association of depression and anxiety with pain: a study from NESDA. Plos One. 2014;9(10):e106907.
19. de Heer EW, ten Have M, van Marwijk HW, Dekker J, de Graaf R, Beekman AT, et al. Pain as a risk factor for common mental disorders. Results from the Netherlands Mental Health Survey and Incidence Study-2: a longitudinal, population-based study. Pain. 2018;159(4):712-8.
20. Veehof MM, Trompetter HR, Bohlmeijer ET, Schreurs KMG. Acceptance- and mindfulness-based interventions for the treatment of chronic pain: A meta-analytic review. Cognitive Behaviour Therapy. 2016;45(1):5-31. PMID: 2016-09638-002. doi: 10.1080/16506073.2015.1098724.
21. Cassidy EL, Atherton RJ, Robertson N, Walsh DA, Gillett R. Mindfulness, functioning and catastrophizing after multidisciplinary pain management for chronic low back pain. Pain. 2012;153(3):644-50.
22. Vowles KE, Witkiewitz K, Sowden G, Ashworth J. Acceptance and commitment therapy for chronic pain: evidence of mediation and clinically significant change following an abbreviated interdisciplinary program of rehabilitation. The Journal of Pain. 2014;15(1):101-13.
