# Supplementary material for: Serious Gaming During Multidisciplinary Rehabilitation for Patients With Chronic Pain or Fatigue Symptoms: Mixed Methods Design of a Realist Process Evaluation
Source: J Med Internet Res. 2020 Mar 9;22(3):e14766. doi: 10.2196/14766 (PMC7091046; doi:10.2196/14766)
Supplement: Multimedia Appendix 2 [file jmir_v22i3e14766_app2.pdf]

Multi-media appendix 2: Additional information about the mindfulness measure

Due to ownership by the rehabilitation centre, information in addition to the following can be provided upon reasonable request:

- Original definitions of the 3 underlying constructs (skills): 'mental stability' (being aware of themselves and being able to focus on daily activities with others), 'enthusiastic perseverance' (to experience joy, take firm decisions, seize opportunities and finish what has been started) and 'patience' (to control the tendency to retaliate, to tolerate, understand and handle bad conditions)
- A total number of 49 5-point Likert-scale items
- All three underlying scales have good internal consistency (Cronbach Alpha >.85).
- Social desirability scores (distance between the average found and the theoretical centre, namely 3, divided by the standard deviation) are all lower than .5.
- In support of construct validity, the following findings correspond with the scales of the five-facet-mindfulness questionnaire (except the 'observe' sub-scale) (see references below);
    - Medium level correlation with psychological well-being (.58 - .69)
    - Medium level correlation with big-5 neuroticism (-.53 - -.59)
    - Very weak to weak correlations with big-5 openness to experience (.14 - .37)
    - Form a common dimension with coping styles (in a joint factor analysis); passive reaction pattern and avoidance [with negative factor loads], and with active styles [with positive factor loads])
    - Weak to medium correlation with depression (-.31 - -.58).

References

Baer, R. A., Smith, G. T., Lykins, E., Button, D., Krietemeyer, J., Sauer, S., ... & Williams, J. M. G. (2008). Construct validity of the five facet mindfulness questionnaire in meditating and nonmeditating samples. *Assessment*, *15*(3), 329-342.

Veehof, M. M., Peter, M., Taal, E., Westerhof, G. J., & Bohlmeijer, E. T. (2011). Psychometric properties of the Dutch Five Facet Mindfulness Questionnaire (FFMQ) in patients with fibromyalgia. *Clinical rheumatology*, *30*(8), 1045-1054.
