# Supplementary material for: Serious Gaming During Multidisciplinary Rehabilitation for Patients With Chronic Pain or Fatigue Symptoms: Mixed Methods Design of a Realist Process Evaluation
Source: J Med Internet Res. 2020 Mar 9;22(3):e14766. doi: 10.2196/14766 (PMC7091046; doi:10.2196/14766)
Supplement: Multimedia Appendix 3 [file jmir_v22i3e14766_app3.pdf]

Multi-media appendix 3: Coding illustrations for individual level intervention - context – mechanism – outcome configurations and barriers

Illustration of the procedure to induct ICMOC's and barriers from individual patient interviews

Step	Coding procedure	Illustration of codes
1	Identify (kinds of) positive, neutral, or negative outcomes as perceived by patients.	'Self-awareness' (O) [Patient 2]: "in your daily life you indeed have to deal with all those facets, even though you are not aware of it, so for awareness it is a very good session to have"
2	Establish 'M-O combinations' by reading through complete patient interviews wherein outcomes were moderately to strongly positive, looking for (explicit language about) reasoning that led to an outcome.	M-O combination of 'learning transfer' (M) and 'self-awareness' (O) [Patient 2]: *"Yes, it is more truly focused on practice. An (information) session is fine: you get the theory, but that does not really contain a link with practice. Often, you miss that. This (LAKA) is very concrete. What I just said: you get that awareness very often in moments of choice in daily life"*
3	Complete ICMOCs for positive outcome expectations from explicit statements on facilitators for M-O combinations.	The previous quote also establishes 'relative advantage' (to other modalities of providing information) (I), and completes the configuration of Self-awareness (O) = 'relative advantage' (I in C) + learning transfer (M)
4	List barriers linked explicitly with negative, neutral, or sub-optimal reasoning or outcome expectations	The following quote establishes flexibility/personalization (I) or patient health status (C) as barriers [Patient 7]: "in light of 'complaints' ... for which I was there, and the process I was in, I felt that LAKA was a bit of a dodge that added little"

Table 2.10: CH6 CMOCs and barriers for self-awareness identified within patient interviews

Resp.	Outcome	Facilitating context and mechanisms or barriers for learning results
1	Present	Resources (intended active ingredients of LAKA, i.e. 'reflections', 'encounters', 'attention exercises', debriefing) & reasoning (confrontations with self, involvement) (M) +<br>Design quality, compatibility, planning (timing when rationale is known) (I in C), patient health status, including concentration problems, tendency to ruminate (C).
2	Present	Resources (debriefing, 'encounters', meditation exercises) & Reasoning (sense of autonomy; reflect on options and consequences, confrontations with self, learning transfer) (M) +<br>Compatibility (of the modality with corresponding approach), relative advantage (to other means of information provision during treatment), inner setting structure (group setting) as facilitated by organizational planning (I in C)<br><br>Barriers: Design quality (too much time is spent on 'fun' parts of the game, planning (timing; closer to 'theoretical sessions') (I in C)
3	Present	Resources (debriefing, encounters, mini-games) & reasoning (confrontations with self, reflect on options and consequences, learning transfer) (M) +<br>Design quality (failure to complete puzzles), personal attributes (highly persistent), inner setting structure (group setting) as facilitated by organizational planning (I in C)<br><br>Barriers: Design feedback provides limited confrontation (with choices), professional (supporting staff) technical expertise and time, facilities and equipment on site (i.e. log-in problems) (I in C)
4	Present	Reasoning (confrontations with self, learning transfer) (M) +<br>Compatibility (of the modality with corresponding approach) and relative advantage (to other means of information provision during treatment) (I in C), and patient attributes (ICT background) (C)<br><br>Barriers: Design feedback provides limited confrontation (with choices), planning (time; too much time between serious gaming and theoretical sessions in program, relative duration / intensity is limited) (I in C), professional (supporting staff) technical expertise

		and time, facilities and equipment on-site (cannot be used with hearing impairment) (C)
5	Present	Reasoning (confrontations with self, sense of autonomy; reflect on options and consequences) (M) +<br>Technical support (2nd session), planning (timing within program) (I in C), patient health status/personal attributes (depressive mood; recurrent) (C)<br><br>Barriers: Design quality (limited adaptability/specificity/flexibility of response options), inner setting structure (group setting) (I in C), provider role perception (1st session), personal attributes (high age, limited experience with technology) (C)
6	Present	Resources ('encounters', meditation exercises) & reasoning (reflect on options and consequences, confrontations with self, learning transfer, involvement, enjoyment) (M) +<br>Compatibility (of the modality with corresponding approach), inner setting structure (group setting) as facilitated by planning (I in C), patient personal attributes (travel experience) and health status (improving and starting to recognize patterns) (C)<br><br>Barriers: Design quality (feedback provides limited confrontation with choices)<br>Planning (too much time between serious gaming and theoretical sessions in program, relative duration / intensity is limited) (I in C)
7	Absent	Barriers: Specificity/flexibility (intervention didn't match personal goals)<br>Design Feedback provides limited confrontation (with choices) (I in C)<br>Patient health status (no difficulties in coping with stress) (C) (barriers)
8	Absent	Barriers: Facilities and equipment on site and no opportunity to play at home, design; feedback provides limited confrontation (with choices) (I in C), professional (supporting staff) technical expertise and time, patient attributes (age, low self-efficacy and high anxiety for technology usage, and negative past experiences) (C)
